# Comparative genomic analysis of Atlantic salmon, *Salmo salar*, from Europe and North America

**DOI:** 10.1186/1471-2156-11-105

**Published:** 2010-11-23

**Authors:** Krzysztof P Lubieniecki, Stacy L Jones, Evelyn A Davidson, Jay Park, Ben F Koop, Seumas Walker, William S Davidson

**Affiliations:** 1 Department of Molecular Biology and Biochemistry, Simon Fraser University, Burnaby, British Columbia, V5A 1S6 Canada; 2 Centre for Biomedical Research, University of Victoria, Victoria, British Columbia, V8W 3N5 Canada; 3 National Institute of Water and Atmospheric Research, Bream Bay Aquaculture Park, PO Box 147, Ruakaka, New Zealand 0151

## Abstract

**Background:**

Several lines of evidence including allozyme analysis, restriction digest patterns and sequencing of mtDNA as well as mini- and micro-satellite allele frequencies indicate that Atlantic salmon (*Salmo salar*) from North America and Europe are genetically distinct. These observations are supported by karyotype analysis, which revealed that North American Atlantic salmon have 27 pairs of chromosomes whereas European salmon have 29 pairs. We set out to construct a linkage map for a North American Atlantic salmon family and to compare this map with the well developed map for European Atlantic salmon.

**Results:**

We used microsatellite markers, which had previously been mapped in the two Atlantic salmon SALMAP mapping families from the River Tay, Scotland, to carry out linkage analysis in an Atlantic salmon family (NB1) whose parents were derived from the Saint John River stock in New Brunswick, Canada. As large differences in recombination rates between female and male Atlantic salmon have been noted, separate genetic maps were constructed for each sex. The female linkage map comprises 218 markers in 37 linkage groups while the male map has 226 markers in 28 linkage groups. We combined 280 markers from the female and male maps into 27 composite linkage groups, which correspond to the haploid number of chromosomes in Atlantic salmon from the Western Atlantic.

**Conclusions:**

A comparison of the composite NB1 and SALMAP linkage maps revealed the reason for the difference in the chromosome numbers between European and North American Atlantic salmon: Linkage groups AS-4 and AS-32 in the Scottish salmon, which correspond to chromosomes Ssa-6 and Ssa-22, are combined into a single NB1 linkage group as are linkage groups AS-21 and AS-33 (corresponding to chromosomes Ssa-26 and Ssa-28). The comparison of the linkage maps also suggested some additional chromosomal rearrangements, but it will require finer mapping, potentially using SNPs, to test these predictions. Our results provide the first comparison of the genomic architecture of Atlantic salmon from North America and Europe with respect to chromosome organization.

## Background

The genus *Salmo *comprises two main species: brown trout (*S. trutta*) and Atlantic salmon (*S. salar*) [1]. Brown trout are native to Europe whereas Atlantic salmon occur naturally on both sides of the Atlantic Ocean. North American and European populations of Atlantic salmon are genetically distinct from one another, with the European populations being further divided into two main groups corresponding to the Baltic drainage and rivers that flow into the Atlantic Ocean [2,3]. This latter genetic difference, while not as substantial as the differentiation between European and North American Atlantic salmon, is greater than the genetic differences that occur between salmon from different rivers and tributaries, which creates thousands of subpopulations [4].

The first study that indicated that there is a genetic difference between Atlantic salmon from Europe and North America involved four electrophoretic alleles of the serum protein transferrin (*Tf*) [5]. *Tf_1 _*is present in all populations, whereas *Tf*_2 _is only present in European Atlantic salmon and the *Tf_3 _*and *Tf_4 _*alleles are restricted to North American salmon. This substantial difference prompted Payne *et al*. [5] to suggest that the European and North American Atlantic salmon should be given subspecies status as *Salmo salar europaeus *and *Salmo salar americanus*, respectively. Allozyme analysis gave similar results for NAD^+^-dependent malic enzyme [6] and malate dehydrogenase, *MDH-3,4 *[7]. Restriction enzyme analysis [8] and direct sequencing [9] of mitochondrial DNA (mtDNA) also demonstrated the genetic differentiation of European and North American Atlantic salmon. It has been estimated that more than 44% of the variation observed in Atlantic salmon mtDNA is due to diagnostic differences that can be used to distinguish European and North American Atlantic salmon [3]. Genetic markers from the nuclear genome, such as a restriction fragment length polymorphism in the ribosomal RNA gene complex (rDNA) [10] and the minisatellite probe *Ssa*-A45/2/2 [11], have also been shown to differentiate Atlantic salmon from both sides of the Atlantic Ocean.

Perhaps the most convincing genetic evidence that North American and European Atlantic salmon are genetically distinct comes from karyotype analysis. Chromosomal rearrangements, particularly Robertsonian fissions and fusions, are common in salmonids [12,13]. The karyotype of Atlantic salmon is variable with chromosome numbers of 53 to 60 being reported [14-16]. Atlantic salmon from Atlantic Canada and Maine generally have 27 chromosome pairs and 72 chromosome arms [3,12] although other combinations have been reported [14,15,17]. In contrast, Atlantic salmon from Europe commonly have 29 pairs of chromosomes with 74 chromosome arms [18,19]. A picture of the karyotype commonly found in European Atlantic salmon can be seen in Figure three of [12] while the karyotype commonly observed in North American Atlantic salmon is shown in Figure five of [13]. A minimum of two Robertsonian fusions or fissions could have produced the difference in the number of chromosomes between North American and European Atlantic salmon; however, it is anticipated that additional chromosomal rearrangements, such as inversions and translocations, have also occurred [3].

Despite the genetic and chromosomal differences between Atlantic salmon from Europe and North America, it has been possible to produce fertile hybrids and viable back-crosses; however, no information about relative survival of the offspring was given in this report [20]. The viability of this hybrid is not unexpected given the apparent plasticity of salmonid genomes and the observation that Atlantic salmon and brown trout (*Salmo trutta*; 2n = 80) produce hybrids in the wild [21] and under aquaculture conditions [22]. Therefore, the definition of species, let alone sub-species, in the salmonid fishes should not rest on their ability or inability to interbreed [23].

Our goal was to compare the genomic architecture of Atlantic salmon from North America and Europe with respect to chromosome organization. We expected that the chromosomal differences between North American and European Atlantic salmon would be reflected in their genetic maps. As the integration of the genetic map and the karyotype of European Atlantic salmon has recently been completed [24], we first constructed a linkage map for an Atlantic salmon aquaculture broodstock family (NB1) whose parents were derived from the Saint John River in New Brunswick, Canada. Then we compared this map with the genetic map produced from the two SALMAP Atlantic salmon mapping families [25] whose parents came from the River Tay in Scotland. It was anticipated that if the maps contain enough markers in common and are sufficiently dense, it would be possible to identify gross differences in linkage groups corresponding to chromosomal alterations. These predicted alterations could subsequently be assessed using fluorescent in situ hybridization (FISH) analysis.

## Results and Discussion

### Microsatellite markers, variation in parents, and pedigree analysis

The parents of the NB1 family were part of a broodstock development program based on Saint John River stock. The mitochondrial genomes of the parents were analyzed, and we found that the mitochondrial DNA of both parents had sequences [9] and haplotypes [8] that are characteristic of North American Atlantic salmon. The parents of the NB1 family were screened with 718 genetic markers, which were chosen based on them having been mapped in the Br5 or Br6 SALMAP Atlantic salmon mapping families [24,25]. The genetic markers are described in Additional file 1, Table S1. Additional information, including primer sequences and GenBank accession numbers or references can be found in the marker database of the Atlantic salmon genomics database [26]. Of the 718 genetic markers, 35 were SNPs identified from ESTs whereas the others were microsatellite markers, seven of which came from ESTs, 634 were derived from BAC end sequences and 42 were isolated from anonymous regions of DNA. The vast majority of the genetic markers were from Atlantic salmon (705) with the others originating from rainbow trout (9), Arctic charr (1), pink salmon (1), chinook salmon (1) and sockeye salmon (1) (Table 1). One hundred and ninety microsatellite primer pairs failed to give a PCR product that could be used for genotyping analysis (i.e., either no product or a series of bands indicating multiple amplification products). It should be noted that we used a standard protocol for amplifying the microsatellite markers [25] and did not seek to optimize conditions for each primer pair. There may be differences in the primer binding sites in the North American individuals compared to their European counterparts, but this probably does not account for all of these failed reactions. It is worth noting that all of the genetic markers derived from ESTs, whether SNPs or microsatellites, gave clean PCR products, which could be used for genotyping purposes. Nine of the non-Atlantic salmon primers from anonymous DNA gave clean PCR products and four gave poor quality products, which was similar to the results for Atlantic salmon primers from anonymous DNA; nine giving clean, usable products and five yielding poor quality products. Two hundred and thirty eight of the 528 genetic markers, which could be scored in the parents of the NB1 family, were monomorphic and thus non-informative for linkage analysis. The observation that 45% of the genetic markers were not variable in both of the NB1 parents suggests that considerable in-breeding had occurred in the Atlantic salmon broodstock development program that produced the NB1 family. Four of the 290 informative markers (i.e., one or both parents were heterozygous for that marker) were duplicate loci as evidenced by the appearance of three or four alleles. The duplicate nature of the markers Ssa0063BSFU, Ssa0067BSFU, Ssa0290BSFU and OMM5037 is identified by a/I or/II after their name on the linkage maps. Therefore, a total of 294 loci were genotyped in the NB1 family and 279 were assigned to the female or male specific linkage maps of the NB1 family.

**Table 1:** Description of markers used in the construction of the linkage map for the NB1 family.

Number of markers	Type of marker	Sequence source	**Species**^**1**^	**Informative**^**2**^**,** Non-informative **or Poor PCR product**^**3**^	Mapped in NB1 family
**17**	SNP	EST	AS	Informative	Yes
**4**	Microsatellite	EST	AS	Informative	Yes
**237**	Microsatellite	BAC end	AS	Informative	Yes
**15**	Microsatellite	Anonymous	AS	Informative	Yes
**4**	Microsatellite	Anonymous	RT	Informative	Yes
**1**	SNP	EST	AS	Informative	No
**12**	Microsatellite	BAC end	AS	Informative	No
**290**					
					
**17**	SNP	EST	AS	Non-informative	No
**3**	Microsatellite	EST	AS	Non-informative	No
**204**	Microsatellite	BAC end	AS	Non-informative	No
**9**	Microsatellite	Anonymous	AS	Non-informative	No
**1**	Microsatellite	Anonymous	AC	Non-informative	No
**1**	Microsatellite	Anonymous	PS	Non-informative	No
**3**	Microsatellite	Anonymous	RT	Non-informative	No
**238**					
					
**181**	Microsatellite	BAC end	AS	Poor PCR product	No
**5**	Microsatellite	Anonymous	AS	Poor PCR product	No
**1**	Microsatellite	Anonymous	CS	Poor PCR product	No
**2**	Microsatellite	Anonymous	RT	Poor PCR product	No
**1**	Microsatellite	Anonymous	SS	Poor PCR product	No
**190**					

Species^1 ^abbreviations are as follows: AS, Atlantic salmon; RT, rainbow trout; AC, Arctic charr; CS, chinook salmon; PS, pink salmon; SS, sockeye salmon. Informative^2 ^and non-informative refers to the markers being heterozygous in one or both of the NB1 parents. Poor PCR product^3 ^means that there was no amplification product or multiple bands that made it impossible to assign a genotype. Note that a standard PCR assay was used for all markers and no optimization was carried out. More details about the markers can be found in Additional file 1, Table S1. The primers for the markers can be found in the marker database of the Atlantic salmon genomics database [26].

### Construction of female-specific and male-specific linkage maps and a composite genetic map for the NB1 family

The transmission of alleles from each parent to the offspring was used to construct female-specific and male-specific genetic maps for family NB1 (Additional file 2, Figure S1; Additional file 3, Figure S2; Additional file 4, Figure S3 and Additional file 5, Figure S4). This was deemed necessary as large differences in recombination frequencies in female and male Atlantic salmon have been observed [27,28]. The phase files generated for the female and male NB1 maps (Additional file 6, Table S2 and Additional file 7, Table S3) were examined, and the gels for any marker that gave an apparent discrepancy (i.e., single locus contributing to two recombination events per linkage group) were reviewed for accuracy in scoring. For example, in the NB1-25 m linkage group, Ssa1000BSFU appears to have recombination events between itself and the markers on either side of it (i.e., a highly localized double recombination, Additional file 7, Table S3). Another example of this was seen with marker Ssa0524BSFU in the same linkage group. However, when the genotyping results were reviewed they were confirmed as originally read. Apart from these instances and linkage group NB1-4/32 m, in which there were two double recombinations, one involving a single marker (Ssa0043BSFU), only single recombinations were seen in male linkage groups. We examined the positions of the recombinations in 21 male linkage groups comprising five or more markers. There was a total of 52 recombinations and 45 (86.5%) of them occurred between a terminal marker and the penultimate marker. Therefore, it appears that there is a strong tendency for recombinations to occur at the ends of linkage groups in the male. When a similar analysis was carried out on the female phase files, there were 25 instances of double recombinations and three triple recombinations. When the recombination position was examined in single recombinations in linkage groups with five or more loci, 65 of 174 (37.1%) involved a terminal marker and the penultimate marker. This is in sharp contrast to what was observed in corresponding male linkage groups, indicating that recombinations in females are more broadly distributed along the chromosomes. However, it will require many more markers (i.e., the construction of dense genetic maps) before the patterns of recombination in male and female Atlantic salmon can be fully ascertained.

The female map, which was constructed using a LOD score of 3, comprises 218 markers in 37 linkage groups. The male map, which was constructed using a LOD score of 4, incorporated 226 markers into 28 linkage groups for the male map. There were fifteen loci that remained unassigned to any linkage group at a LOD score of 3 for the female map and a LOD score of 4 for the male map. The length of the female NB1 genetic map is 1143.75 cM whereas the length of the male NB1 genetic map is 314.02 cM. The greater length of the female map compared to the male map reflects the difference in recombination rates between the two sexes, which has been described previously [27,28]. We combined 280 markers from the female and male maps into 27 composite linkage groups, which correspond to the number of haploid chromosomes seen in Atlantic salmon from North America [3].

### Comparison of NB1 composite linkage groups with the European Atlantic salmon genetic map

The NB1 genetic map is derived from 40 offspring of a single pair mating, and therefore, the power to detect linkage is limited. For example, if only one parent is heterozygous for a marker, the power to detect linkage at 5 cM is 90% but only 63% at 10 cM and 18% at 20 cM. Accordingly the largest inter-marker distances observed in the NB1 map are about 20-23 cM (NB1-2f, NB1-15f, NB1-31f) (see Additional file 2, Figure S1; Additional file 3, Figure S2; Additional file 4, Figure S3 and Additional file 5, Figure S4). This lack of power to detect linkage is aptly demonstrated by the initial inability to identify NB1-19f using a LOD score of 3 (see below). However, given that the European Atlantic salmon genetic map is based on two SALMAP families with ~46 offspring in each [24,25], we believe that the NB1 genetic map provides a good preliminary foundation for the comparison of the gross organization of the European and North American Atlantic salmon genomes. Moreover, as additional markers are placed on the map, linkages between distant markers will become evident as will their relative positions.

Additional file 2, Figure S1; Additional file 3, Figure S2; Additional file 4, Figure S3 and Additional file 5, Figure S4 show the integration of the male-specific and female-specific linkage groups from the NB1 family and their comparison with the corresponding linkage groups in the SALMAP merged female map [24-26]. We named the NB1 linkage groups in accordance with the European Atlantic salmon SALMAP family linkage groups taken from the merged female map [24-26] based on the presence of markers in common. Note that there are 29 SALMAP family linkage groups, designated AS-X, and for historical reasons there is no AS-26, AS-27, AS-29 or AS-30. If more than one linkage group in the female or male NB1 family had markers from a single SALMAP linkage group, the NB1 linkage groups were assigned the AS number followed by a letter. For example, markers from AS-10 were located in two of the NB1 male linkage groups (NB1-10am and NB1-10bm) as well as two of the NB1 female linkage groups (NB1-10af and NB1-10bf). In two instances markers from two SALMAP linkage groups (AS-4 and AS-32, and AS-21 and AS-33) were located in a single NB1 male linkage group. We depicted these by NB1-4/32 m and NB1-21/33 m, respectively.

The relationships between the European SALMAP linkage groups and those from the North American NB1 male and female genetic maps are shown in Table 2. All linkage groups from the SALMAP merged female genetic map [24-26] could be matched up with a corresponding linkage group from the NB1 male map (Table 2). We noted that there was no equivalent to AS-19 in the NB1 female map. However, when the female map was constructed using a LOD score of 2 rather than 3, Ssa0504BSFU and Ssa0144BSFU, which are located 59.8 cM from one another in AS-19f, were linked at a genetic distance of 26 cM. Of the six markers that were examined from AS-19, these two were the only ones that were informative in the female parent of NB1.

**Table 2:** European Atlantic salmon SALMAP linkage groups and chromosomes and the corresponding linkage groups from the North American Atlantic salmon NB1 male-specific (LOD score 4) and female-specific (LOD score 3) genetic maps.

European SALMAP Salmon Linkage **Groups (AS-) and Chromosomes (Ssa-)**^**1**^	North American NB1 Male-specific Linkage Groups (NB1-m)	North American NB1 Female-specific Linkage Groups (NB1-f)
AS-1 Ssa-2	NB1-1m	NB1-1af, NB1-1bf
AS-2 Ssa-10	NB1-2m	NB1-2af, NB1-2bf
AS-3 Ssa-14	NB1-3m	NB1-3f
AS-4 Ssa-6	*NB1-4/32m	NB1-4f
AS-5 Ssa-13	NB1-5m	NB1-5f
AS-6 Ssa-12	NB1-6m	NB1-6f
AS-7 Ssa-24	NB1-7m	NB1-7f
AS-8 Ssa-15	NB1-8m	NB1-8f
AS-9 Ssa-11	NB1-9m	NB1-9f
AS-10 Ssa-9	NB1-10am, NB1-10bm	NB1-10af, NB1-10bf
AS-11 Ssa-3	NB1-11m	NB1-11f
AS-12 Ssa-5	NB1-12m	NB1-12af, NB1-12bf, NB1-12cf
AS-13 Ssa-19	NB1-13m	NB1-13f
AS-14 Ssa-21	NB1-14m	NB1-14f
AS-15 Ssa-27	NB1-15m	NB1-15f
AS-16 Ssa-18	NB1-16m	NB1-16f
AS-17 Ssa-1	NB1-17m	NB1-17af, NB1-17bf, NB1-17cf
AS-18 Ssa-23	NB1-18m	NB1-18f
AS-19 Ssa-8	NB1-19m	***NB1-19f
AS-20 Ssa-25	NB1-20m	NB1-20f
AS-21 Ssa-26	**NB1-21/33m	NB1-21f
AS-22 Ssa-17	NB1-22m	NB1-22f
AS-23 Ssa-16	NB1-23m	NB1-23af, NB1-23bf
AS-24 Ssa-7	NB1-24m	NB1-24f
AS-25 Ssa-20	NB1-25m	NB1-25af, NB1-25bf
AS-28 Ssa-4	NB1-28m	NB1-28f
AS-31 Ssa-29	NB1-31m	NB1-31f
AS-32 Ssa-22	*NB1-4/32m	NB1-32f
AS-33 Ssa-28	**NB1-21/33m	NB1-33f

^1^Note that there are 29 European linkage groups, and for historical reasons there is no AS-26, As-27, AS-29 or AS-30. The integration of the European Atlantic salmon SALMAP linkage groups and chromosomes is taken from Phillips *et al*. [24]. The NB1 male-specific linkage groups that correspond to pairs of the SALMAP linkage groups are shown by * and **. ***NB1-19f comprises two markers 26 cM apart, and was only obtained at a LOD score of 2 rather than 3.

We were intrigued by the splitting of AS-10f into two linkage groups in the male and female NB1 maps. At first glance this suggested that the large acrocentric chromosome (Ssa-9) corresponding to AS-10 in European Atlantic salmon corresponds to two chromosomes in North American Atlantic salmon. Chromosome Ssa-9 contains two blocks of intra-chromosomal heterochromatin which divides the chromosome into three sections, each of which correspond to a chromosome/linkage group in rainbow trout [24]. This has been taken as evidence that Ssa-9 is the result of two fusions of ancestral salmonid acrocentric chromosomes [20]. Therefore, it was possible that one of these fusions had occurred in the European Atlantic salmon lineage after it separated from the North American Atlantic salmon. However, when we inspected the NB1-10ma, NB1-10mb, NB1-10fa and NB1-10fb linkage groups, we found that the distribution of the genetic markers in the male and female linkage groups did not support this hypothesis. For example, there are five genetic markers shared between the NB1-10f and NB1-10 m linkage groups. Of the four common markers that are in linkage group NB1-10ma, two (Ssa0042BSFU and Ssa1070BSFU) map to NB1-10fa while the other two (Ssa0885BSFU and Ssa0850BSFU) map to NB1-10fb. The fifth marker in common (SSOSL85) occurs in NB1-10mb and NB1-10fb. Therefore, these results indicate that there should be a single NB1-10 linkage group in the male and female maps. These linkage groups did not come together when a LOD score of 2 or 3 was used to construct the female or male maps, respectively. As more markers are placed on the NB1 linkage maps, we predict that they will allow the joining of the NB1-10ma and Nb1-10mb as well as the NB1-10fa and NB1-10fb linkage groups.

### Prediction of rearrangements in the Western Atlantic salmon chromosomes relative to those of European Atlantic salmon

**Figure 1:**
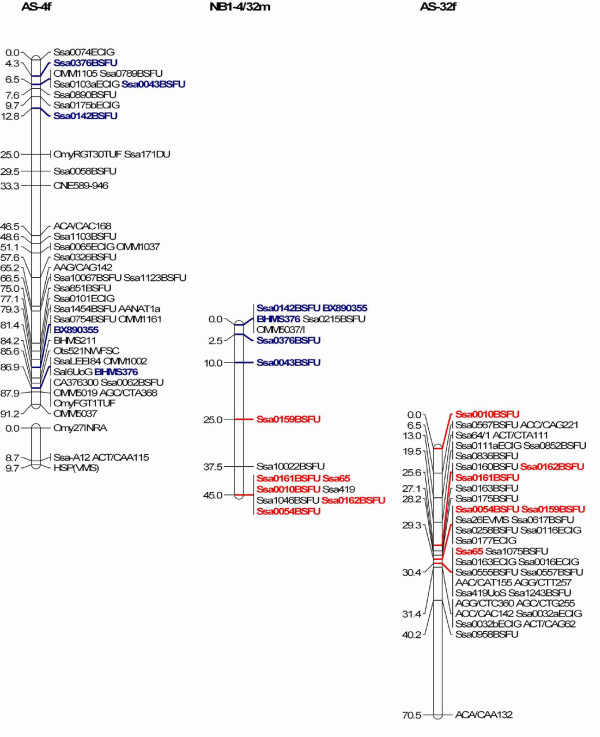
**Comparison of a single linkage group in the NB1 family (NB1-4/32m) with two corresponding linkage groups in the SALMAP integrated female map (AS-4f and AS-32f)**.

**Figure 2:**
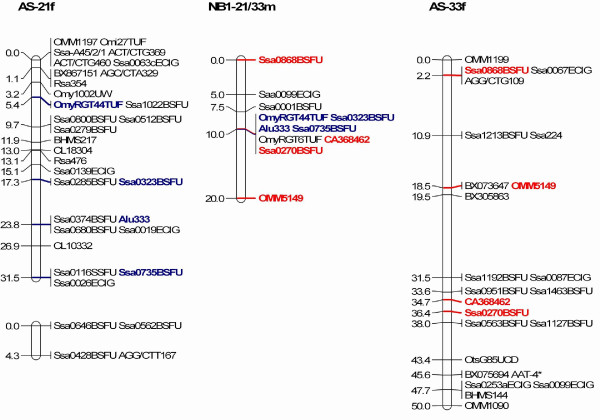
**Comparison of a single linkage group in the NB1 family (NB1-21/33m) with two corresponding linkage groups in the SALMAP integrated female map (AS-21f and AS-33f)**.

The simplest explanation for the difference in the number of chromosomes between the Atlantic salmon from either side of the Atlantic Ocean is that two Robertsonian fissions or fusions having occurred in the European Atlantic salmon (29 chromosome pairs) or North American Atlantic salmon (27 chromosome pairs), respectively. A comparison of the SALMAP composite female linkage map and the composite NB1 genetic map identified two pairs of linkage groups in the SALMAP composite female linkage map that correspond to single linkage groups in the NB1 family. Microsatellite markers in the SALMAP linkage groups AS-4 and AS-32 mapped to a single NB1 male linkage group, NB1-4/32 m (Figure 1). Similarly, markers from SALMAP linkage groups AS-21 and AS-33 mapped to a single male linkage group, NB1-21/33 m (Figure 2). No linkage between NB1-4f and NB1-32f or NB1-21f and NB1-33f was supported when the female NB1 map was constructed using a LOD score of 2. It is unfortunate that the linkages between NB1-4/32 and NB1-21/33 were only made through male analyses and until female linkage is observed, recombination between homeologous chromosomes and potential pseudolinkage effects must be considered as possible explanations. To date, pseudolinkage and all forms of residual tetrasomy have only been observed in male salmonids [29-31].

We examined AS-4 and AS-32 as well as AS-21 and AS-33 for duplicated markers [24,25]. AS-4 shares nine markers with AS-11 and one with AS-8 and AS-10, but there is no evidence for any in common with AS-32. Similarly, AS-32 shares two markers with AS-6 and one with AS-21 but none with AS-4. Therefore, there is no evidence to suggest that AS-4 and AS-32 are homeologs. AS-21 shares a single marker with AS-9 whereas AS-33 has a common marker with AS-31 and AS-17. Therefore, there is no evidence to support AS-21 and AS-33 being homeologs. Pseudolinkage, which is characterized by an unusual marker segregation pattern whereby recombinant progeny types are produced in excess of parental ones during meiosis [32], appears to be minimal in Atlantic salmon compared to what is observed in brown trout and rainbow trout, possibly as a result of more extensive chromosome arm rearrangements limiting the occurrence of multivalent formation in Atlantic salmon [25]. Indeed, only one of the males in the SALMAP European Atlantic salmon mapping families showed a single pseudolinkage grouping, AS-5 and AS-18, [25]. We conclude that the linkage groups NB1-4/32 m and NB1-21/33 m are robust and strongly indicate that they correspond to fusions of the corresponding linkage groups/chromosomes in European Atlantic salmon. Confirmation of these results will require the mapping of additional markers, which may enable the female linkage groups to be joined, or FISH analyses using BAC clones that contain specific genetic markers.

The results described above provide an explanation why the SALMAP genetic map has 29 linkage groups whereas the NB1 genetic map has 27. As the linkage groups and chromosomes have been integrated in European Atlantic salmon [24] it was possible to predict which chromosomes would be fused/rearranged in the North American Atlantic salmon relative to their European Atlantic salmon counterparts. The SALMAP linkage group AS-32 corresponds to an acrocentric chromosome, Ssa-22, while AS-4 corresponds to a metacentric chromosome, Ssa-6 [24]. A fusion of the centromere of Ssa-22 with a telomere of Ssa-6 would produce a metacentric chromosome with an additional heterochromatin block that would be the remnant of the second centromere in the recombinant chromosome. We predict that this recombinant chromosome corresponds to chromosome 6 in the North American Atlantic salmon karyotype illustrated in King *et al*. [3]. AS-21 and AS-33 both correspond to acroentric chromosomes, Ssa-26 and Ssa-28, respectively [24]. The simplest fusion of these chromosomes would be through their centromeres, which would produce a metacentric chromosome. However, the comparison of the NB1-21/33 m with AS-21 and AS-33 (Figure 2) suggests that after the fusion of Ssa-26 and Ssa-28 through their centromeres, there was a pericentric inversion such that in the recombinant North American Atlantic salmon chromosome, markers located on AS-33 flank markers that are seen in AS-21. An alternative scenario is that there was a centromere to telomere fusion followed by a pericentric inversion. In either of these cases, we predict that this series of events would have produced a metacentric chromosome in North American Atlantic salmon that contains the genetic material from two acrocentric chromosomes in European Atlantic salmon. These rearrangements of the European Atlantic salmon chromosomes would change the composition of the karyotype from eight metacentics plus twelve large acrocentrics and nine small acrocentrics to nine metacentrics plus twelve large acrocentrics and six small acrocentrics as has been described for the karyotype of North American Atlantic salmon [3]. By using fluorescent in situ hybridization analysis with selected BAC clones, it should be possible to determine if these predictions are correct and which chromosomal regions in the Western Atlantic salmon chromosomes correspond to the well-defined segments of the European salmon chromosomes [24].

The comparison of the NB1 composite linkage groups with the SALMAP female composite genetic map also suggested that some other rearrangements had occurred. For example, the order of markers at the top of AS-25f is inverted relative to those markers in NB1-25 m, suggesting that there is an inversion in the equivalent of the European Atlantic salmon acrocentric chromosome Ssa-20 in the North American Atlantic salmon karyotype. However, the relatively small number of molecular markers in the NB1 genetic map and the limited power of the NB1 family to detect linkage (see above) precludes making accurate predictions of this and other rearrangements. This will have to await the production of denser genetic maps, which will become available using an Atlantic salmon SNP microarray [33].

## Conclusions

Here we report the first genetic map for North American Atlantic salmon. A comparison of this map with the corresponding genetic map for European Atlantic salmon led us to propose testable predictions about the evolution of chromosome number and a rationale for the gross chromosomal differences seen between Atlantic salmon from both sides of the Atlantic Ocean. It is somewhat surprising that, despite their chromosomal differences, North American and European Atlantic salmon produce viable, fertile hybrids [20]. The difference in karyotypes has been taken as support for the classification of European and North American Atlantic salmon as separate sub-species: *Salmo salar europeaus *and *Salmo salar americanus*, respectively [3,5], although this nomenclature has been criticized for not following standard taxonomic nomenclature [34].

## Methods

### NB1 mapping family and DNA isolation

The NB1 mapping family consists of two parents, derived from the Saint John River, New Brunswick, Canada and 40 offspring. The family, which was part of a broodstock development program, was produced in the fall of 2005, and was maintained at Fisheries and Oceans Canada, St. Andrews Biological Station, St. Andrews, New Brunswick. Tissue samples from the parents were obtained at time of spawning. Blood samples were collected from the offspring in the fall of 2006. The offspring were not euthanized at the time of blood collection, and unfortunately it was not possible to determine their gender from external morphological characteristics. DNA was isolated from the blood samples using the PUREGENE™ DNA isolation kit protocol for "DNA isolation from 2 μL Non-Mammalian Whole Blood" (QIAGEN Inc., Mississauga, Ontario).

### Microsatellite analysis

Microsatellite analysis was carried out according to the methods used to construct the European SALMAP Atlantic salmon linkage group [25]. Information concerning the microsatellite markers used in this study, including the sequences of the primers, can be found in the markerdb section of the Atlantic salmon genomics database [26] or can be obtained from the corresponding author (WSD) upon request.

### Linkage map construction

The genotypes for the parents and offspring were entered into the LINKMFEX software http://www.uoguelph.ca/~rdanzman/software/LINKMFEX/[35], which was used to analyze the genotypes and generate linkage maps for both the female and male parents of the NB1 family at a LOD score of 3 and 4, respectively. The figures showing the linkage groups were prepared using MAPCHART [36]. Genome length and map coverage were determined as described by Fishman *et al*. [37].

## Authors' contributions

WSD and BFK conceived and designed the project. SJ, EAD and JP carried out the genotyping. SJ and KPL constructed the NB1 linkage maps and made the comparisons with the SALMAP maps. SW was responsible for producing and rearing the NB1 family. KPL, SJ and WSD prepared the manuscript and the figures. All authors commented on drafts of the manuscript and approved the final version.

## Supplementary Material

Additional file 1 **Table S1 Information on the genetic markers used in this project**. Click here for file

Additional file 2 **Figure S1 Comparison of the merged SALMAP female linkage groups with the corresponding male-specific and female-specific linkage groups from the NB1 family**. Click here for file

Additional file 3 **Figure S2 Continuation of the comparison of the merged SALMAP female linkage groups with the corresponding male-specific and female-specific linkage groups from the NB1 family**. Click here for file

Additional file 4 **Figure S3 Continuation of the comparison of the merged SALMAP female linkage groups with the corresponding male-specific and female-specific linkage groups from the NB1 family**. Click here for file

Additional file 5 **Figure S4 Continuation of the comparison of the merged SALMAP female linkage groups with the corresponding male-specific and female-specific linkage groups from the NB1 family**. Click here for file

Additional file 6 **Table S2 Phase files for NB1 female-specific linkage groups**. Click here for file

Additional file 7 **Table S3 Phase files for NB1male-specific linkage groups**. Click here for file
