# Supplementary figures and images for: Comparative genomic analysis of Atlantic salmon, Salmo salar, from Europe and North America
Source: BMC Genet. 2010 Nov 23;11:105. doi: 10.1186/1471-2156-11-105 (PMC2995484; doi:10.1186/1471-2156-11-105)

## Slide 1
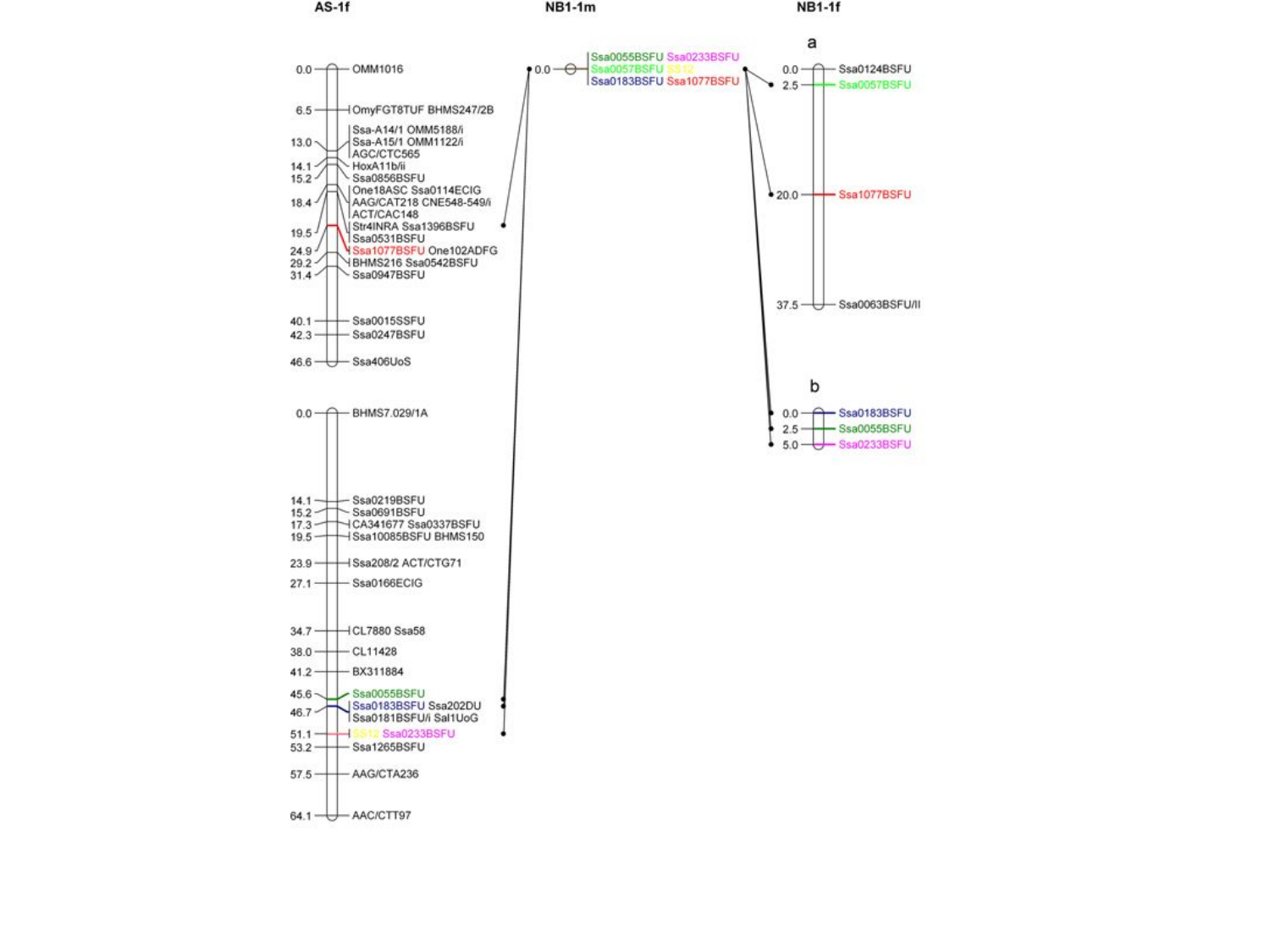

## Slide 2
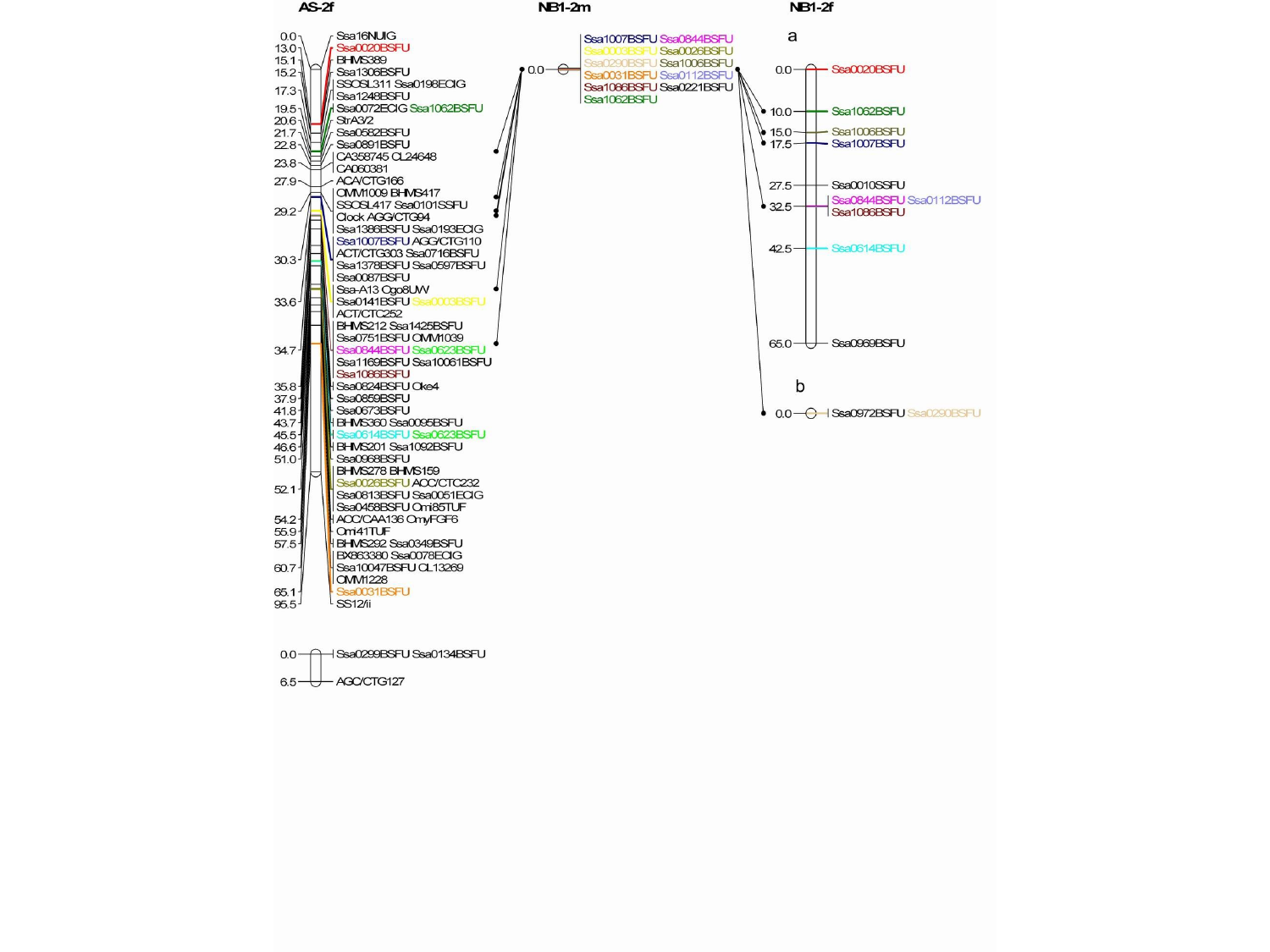

## Slide 3
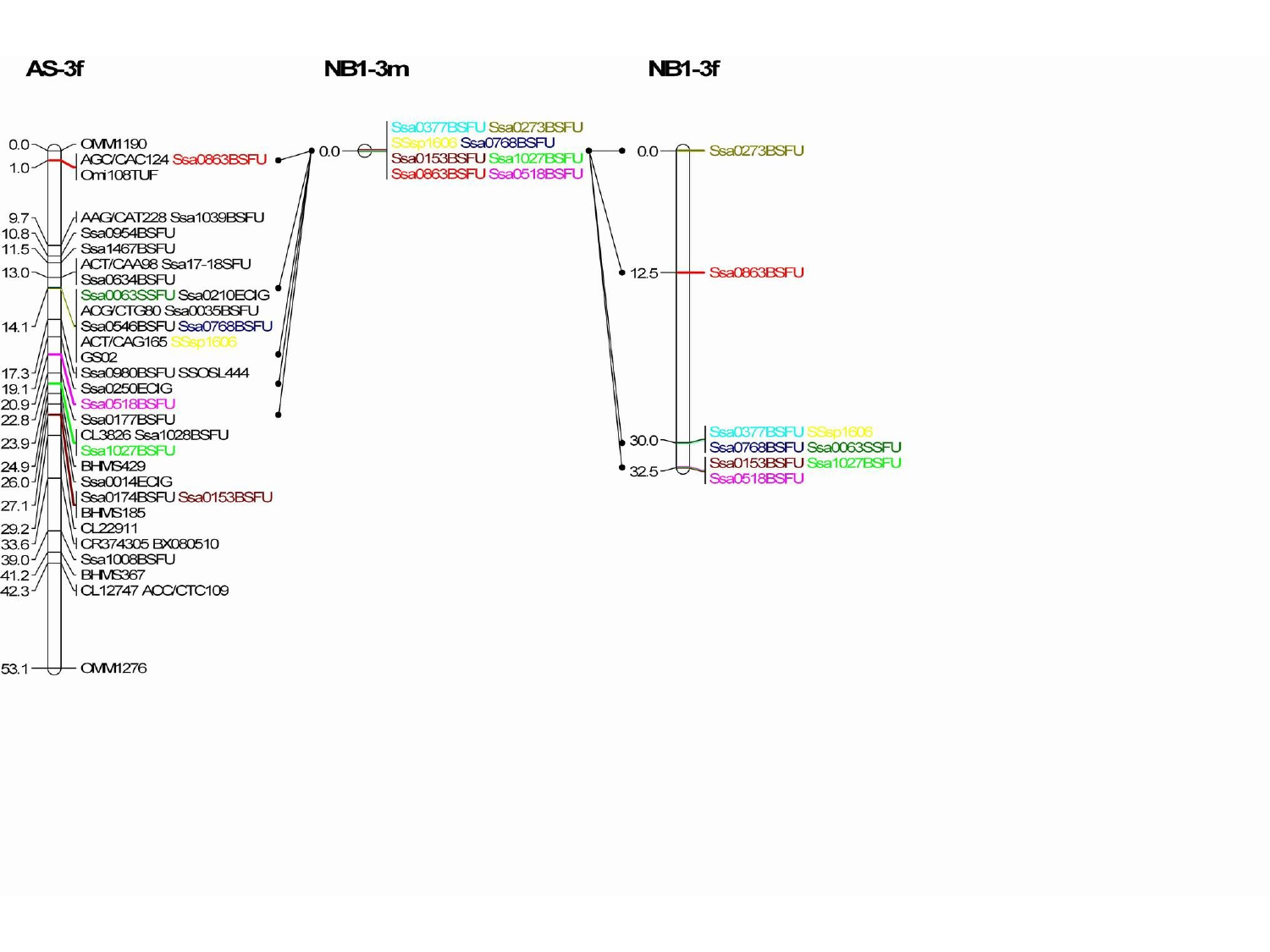

## Slide 4
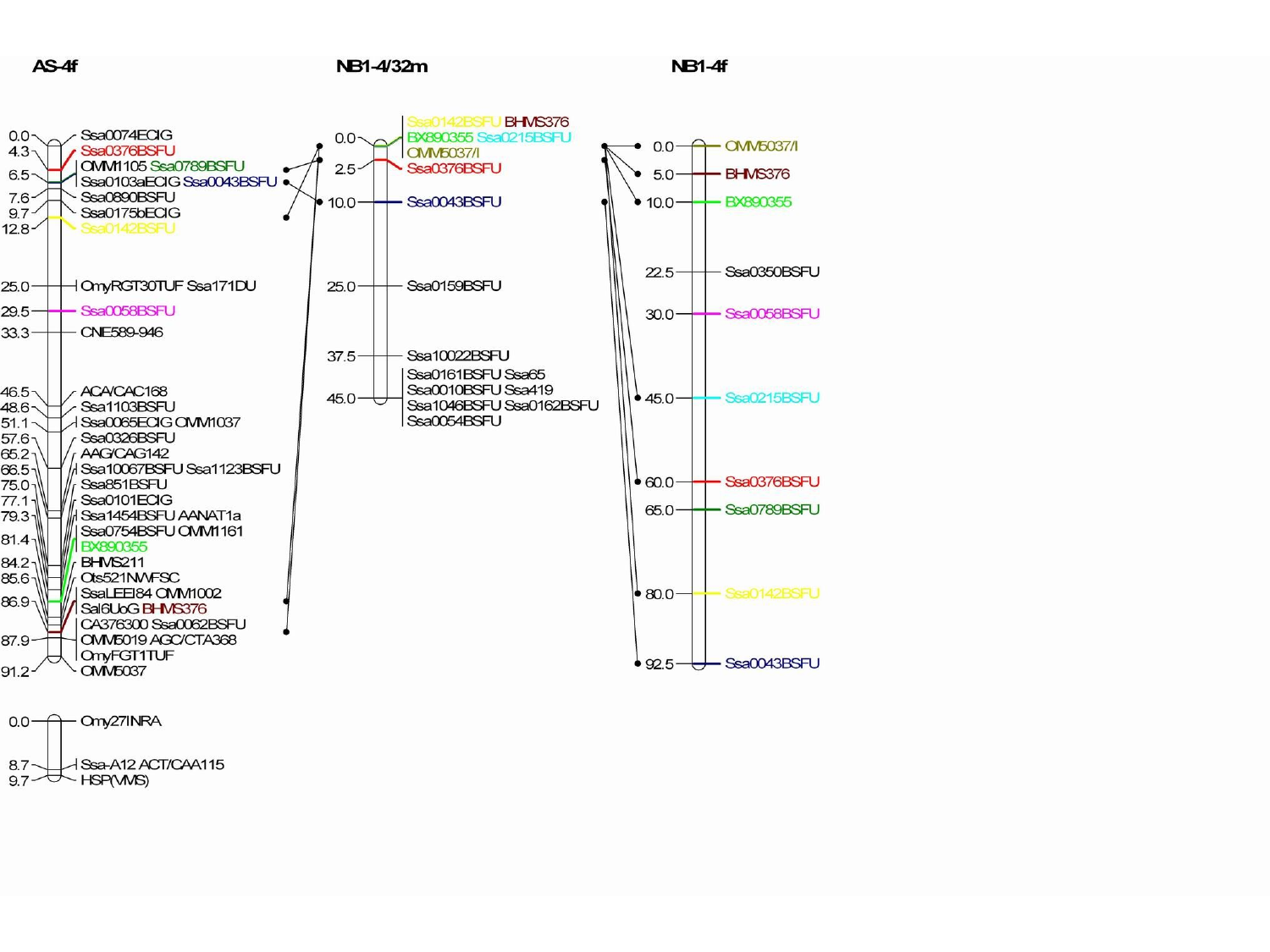

## Slide 5
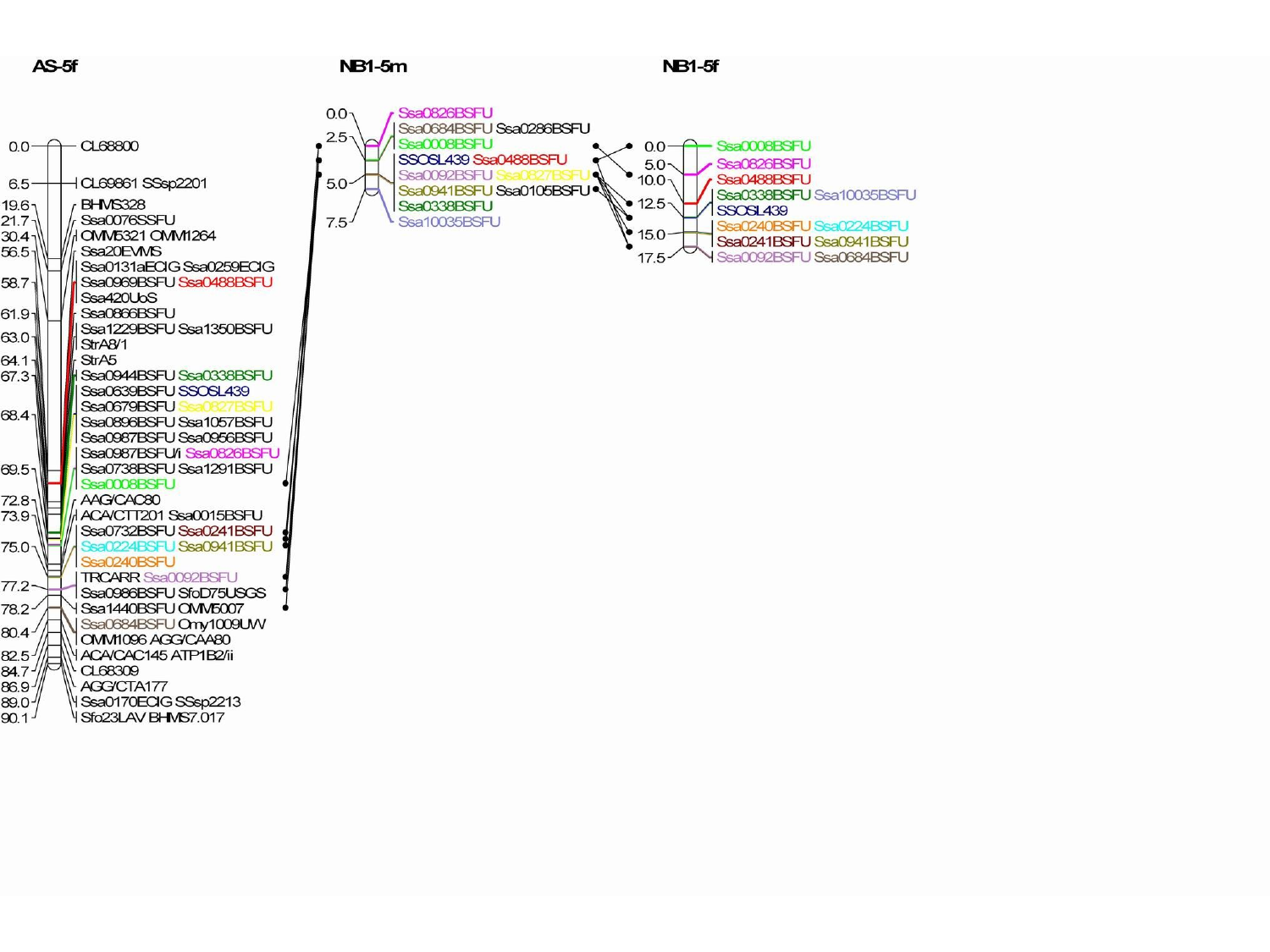

## Slide 6
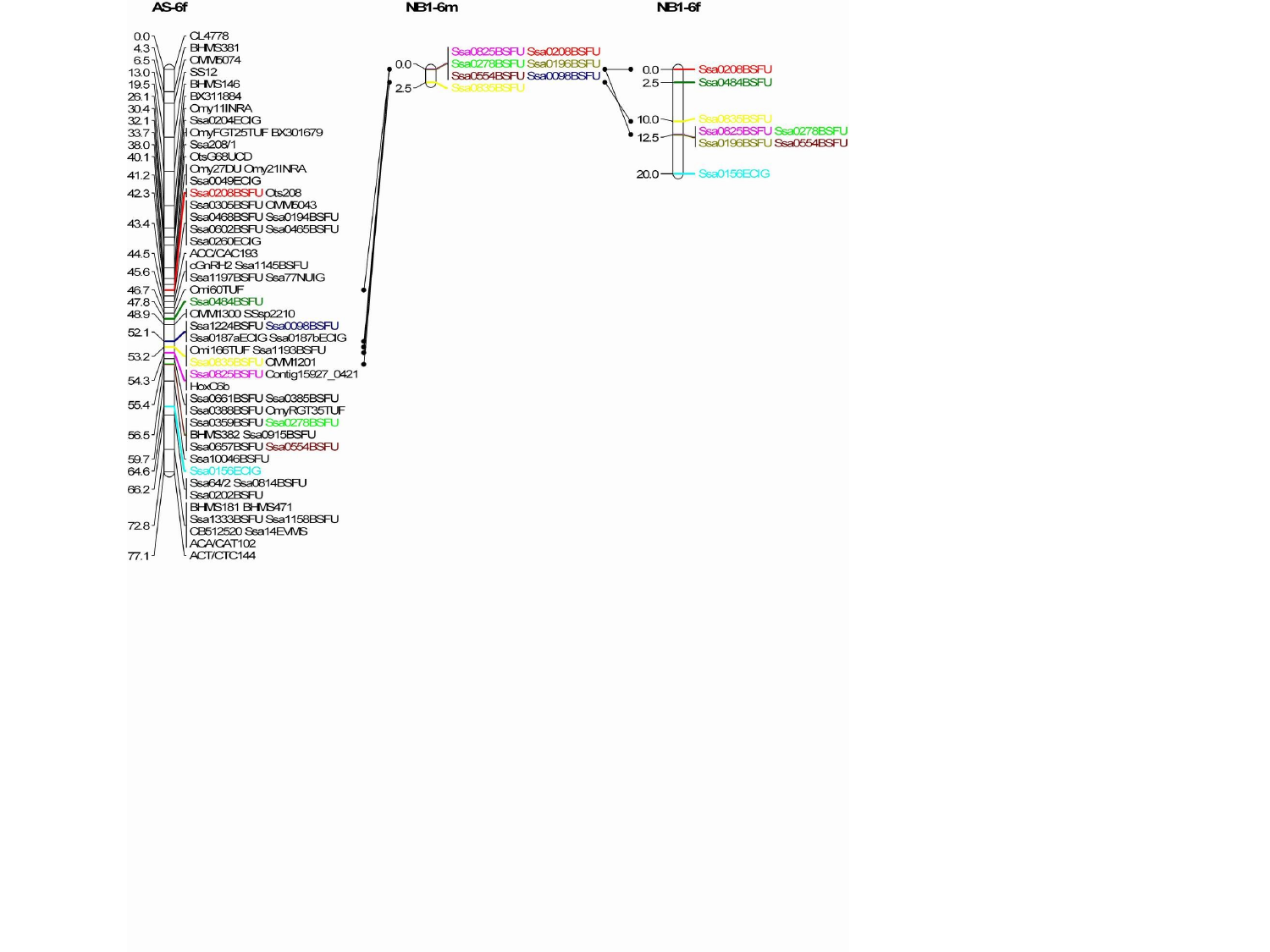

## Slide 7
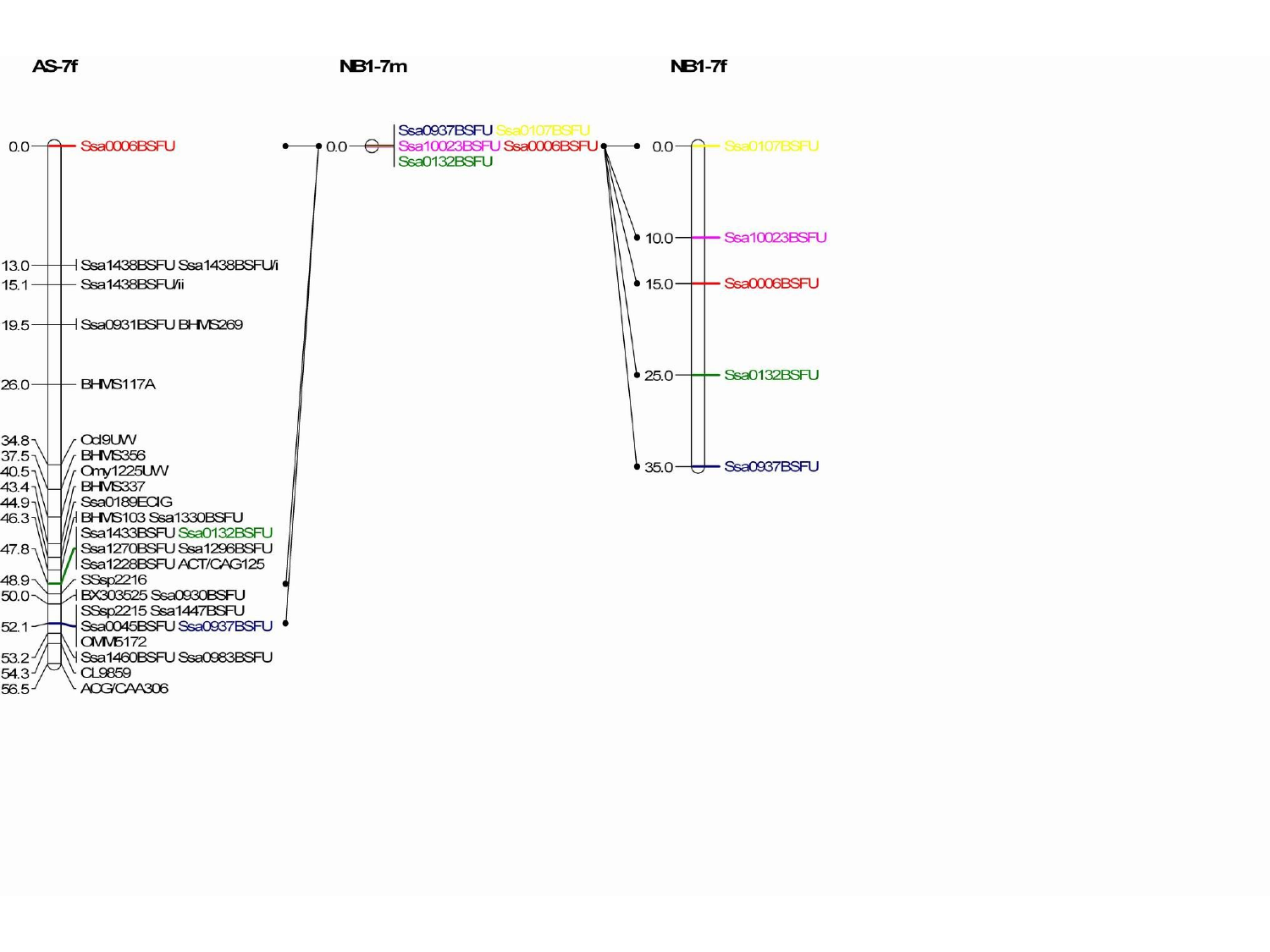

## Slide 8
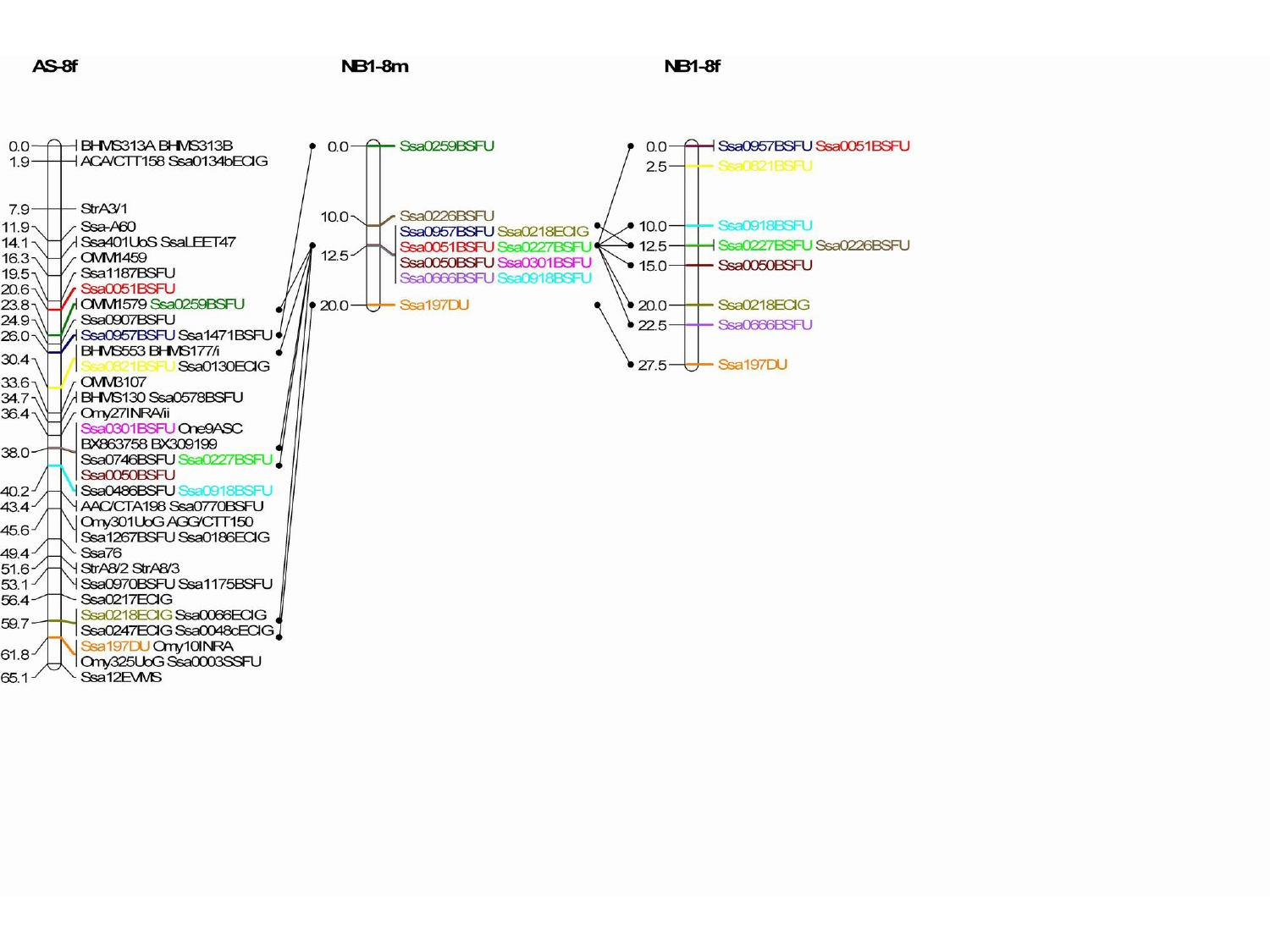

Supplement: Additional file 2 — Figure S1 Comparison of the merged SALMAP female linkage groups with the corresponding male-specific and female-specific linkage groups from the NB1 family. [file 1471-2156-11-105-S2.PPTX]

## Slide 1
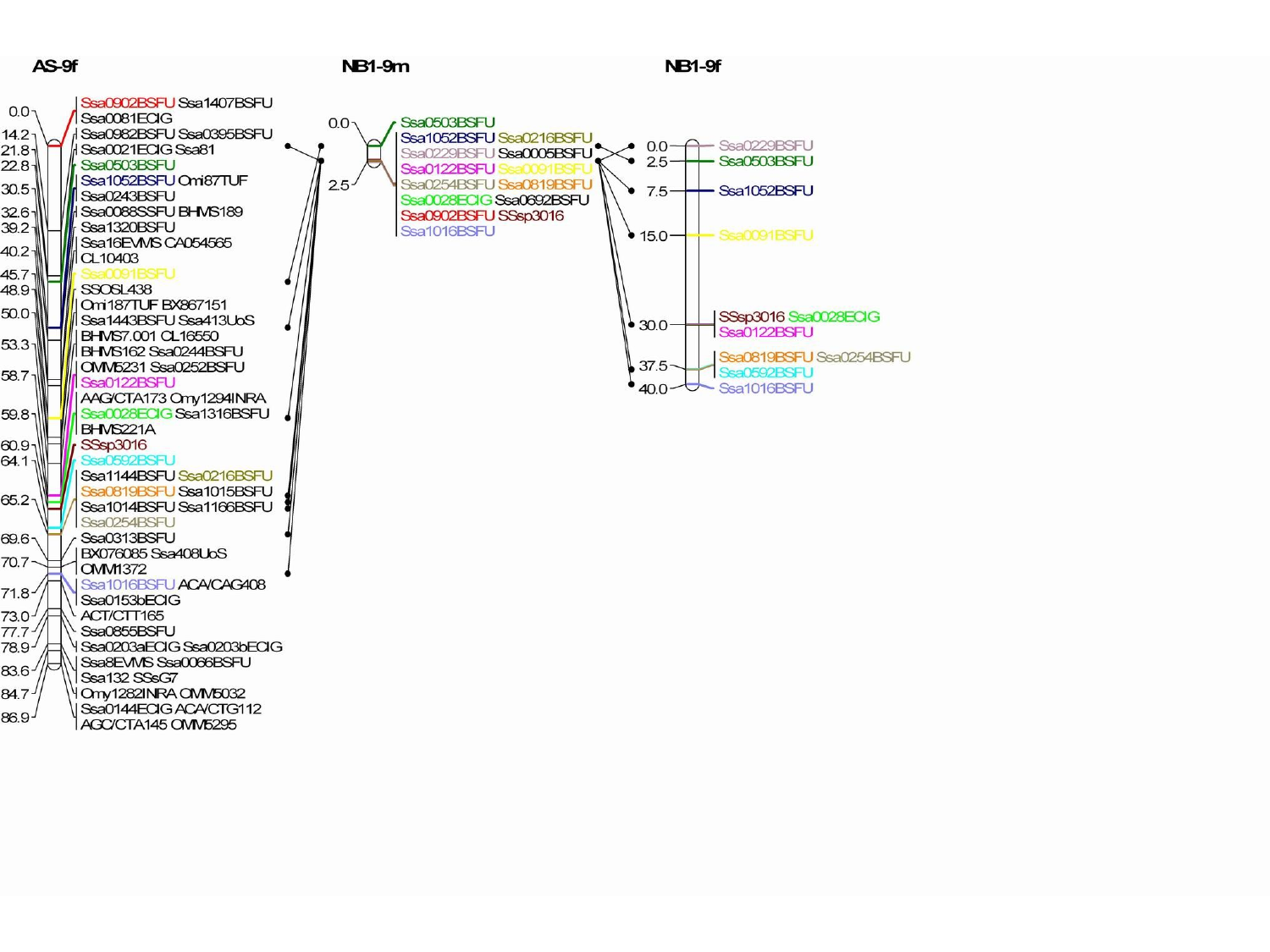

## Slide 2
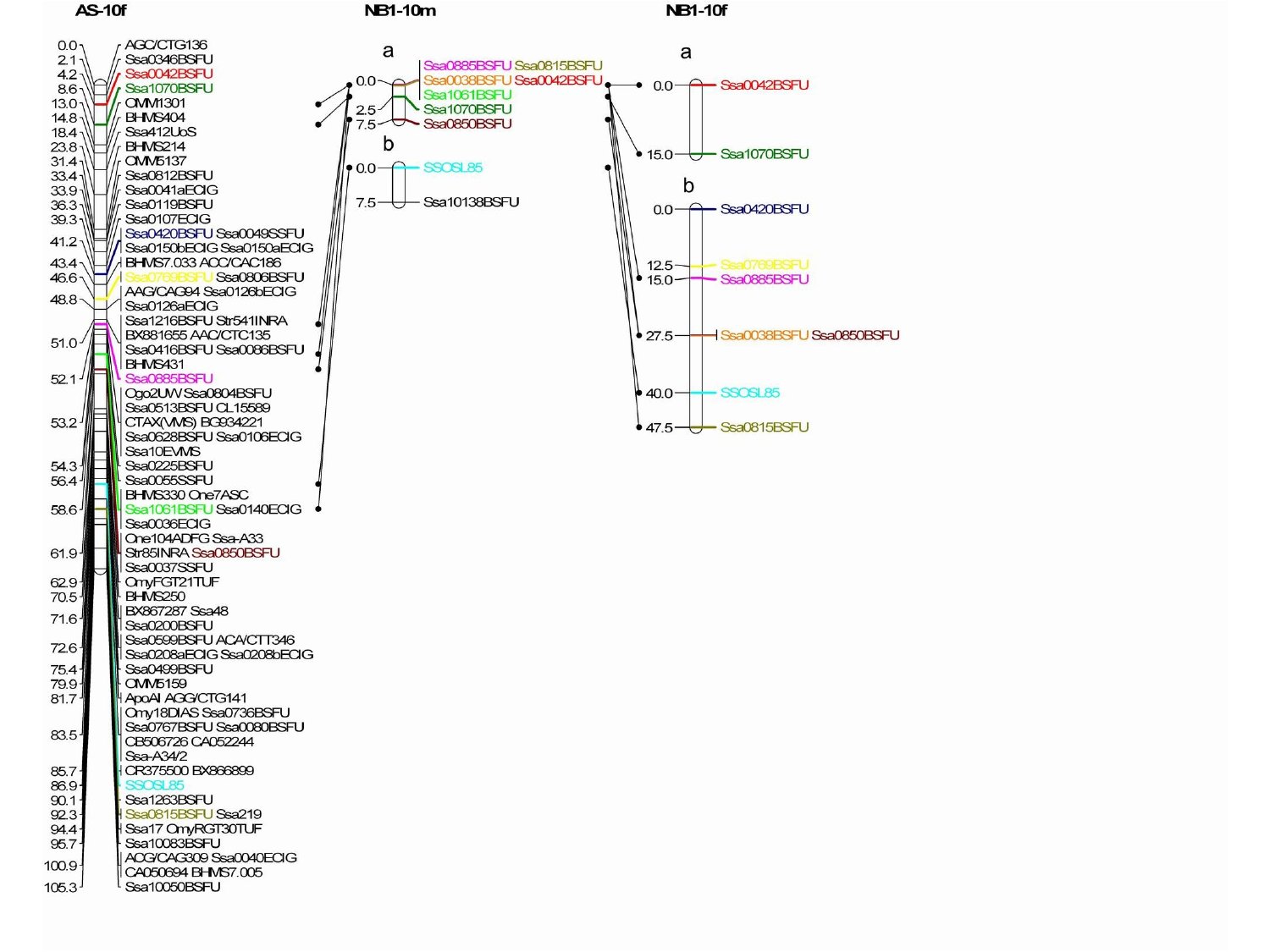

## Slide 3
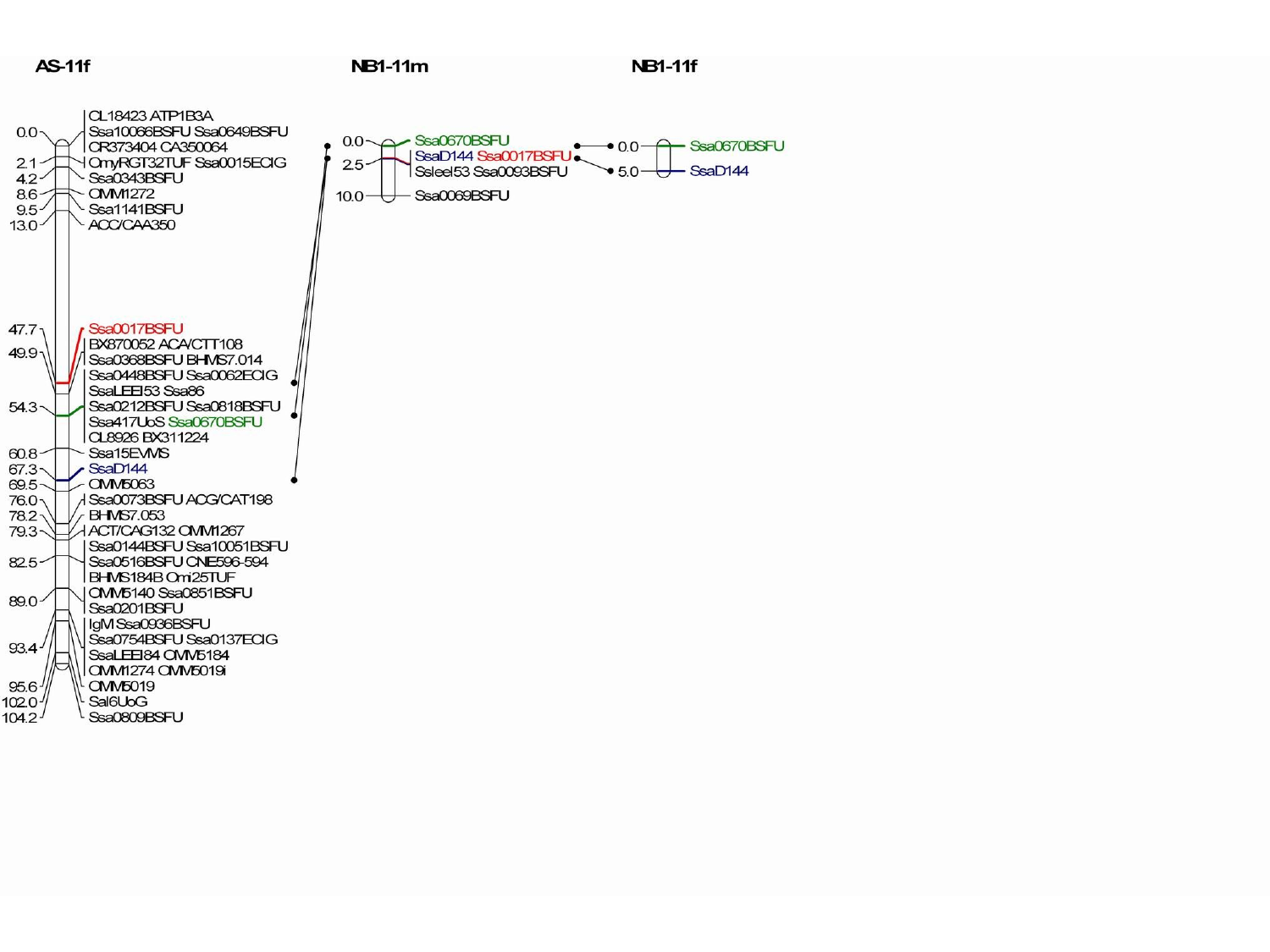

## Slide 4
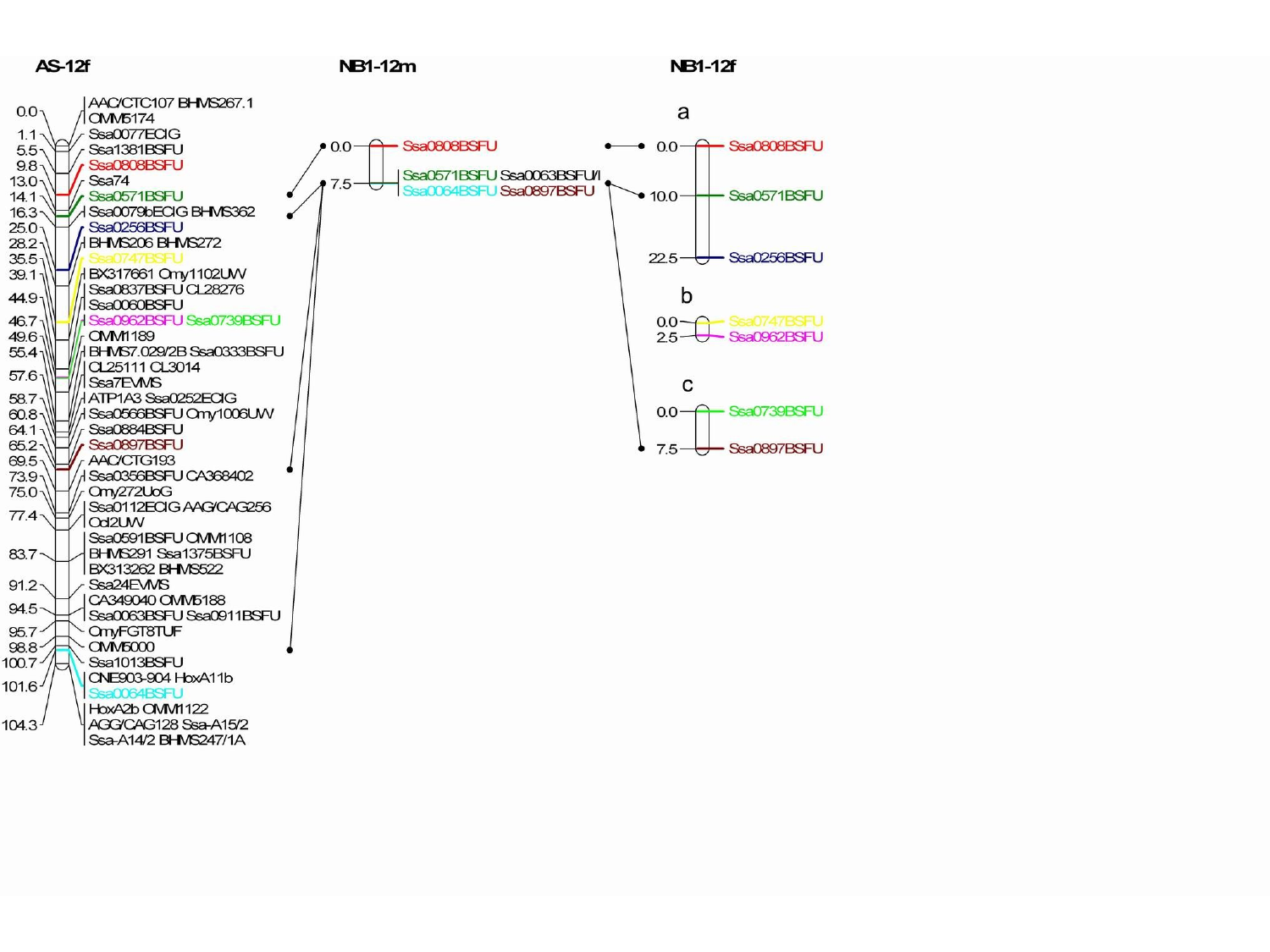

## Slide 5
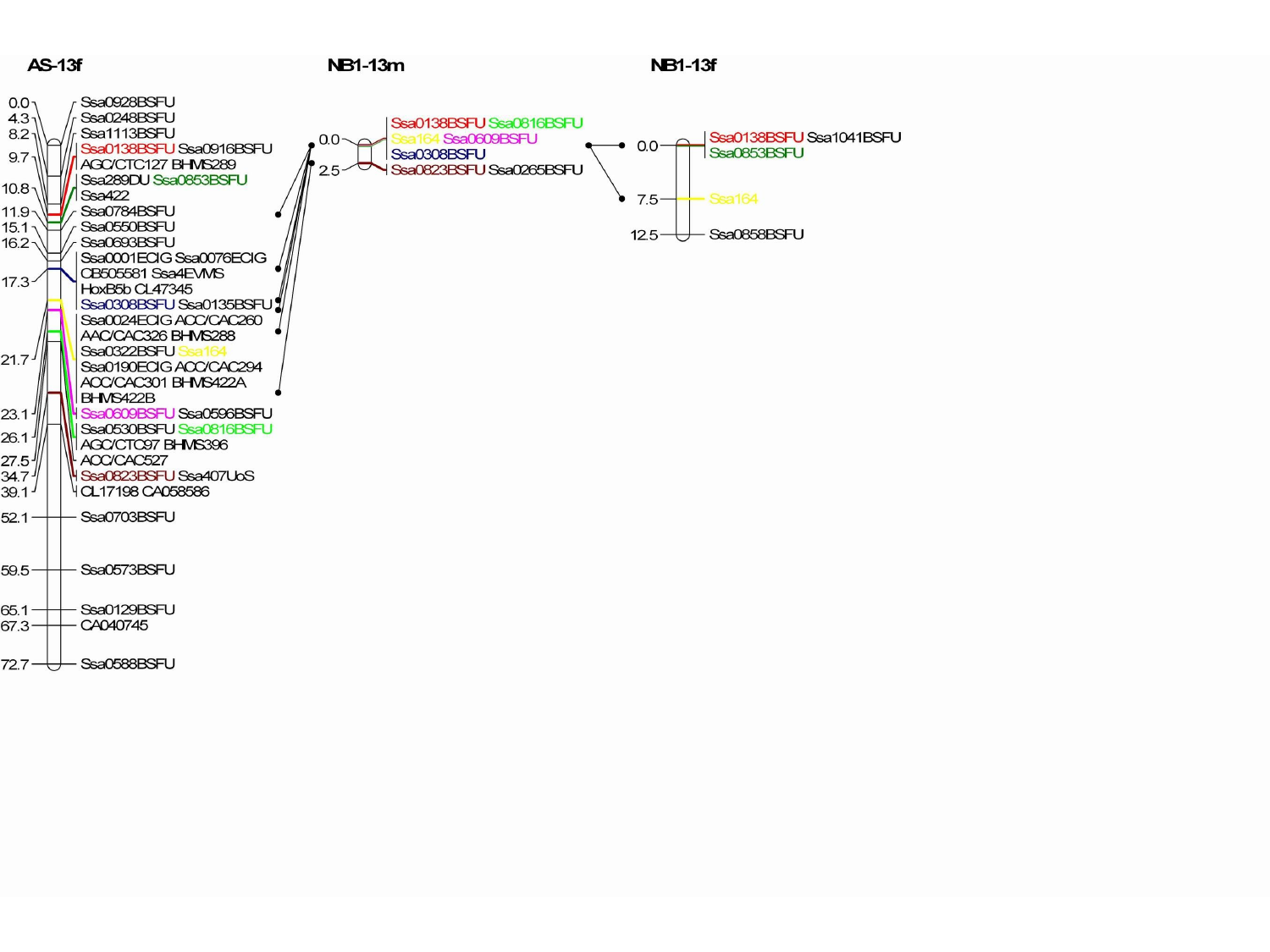

## Slide 6
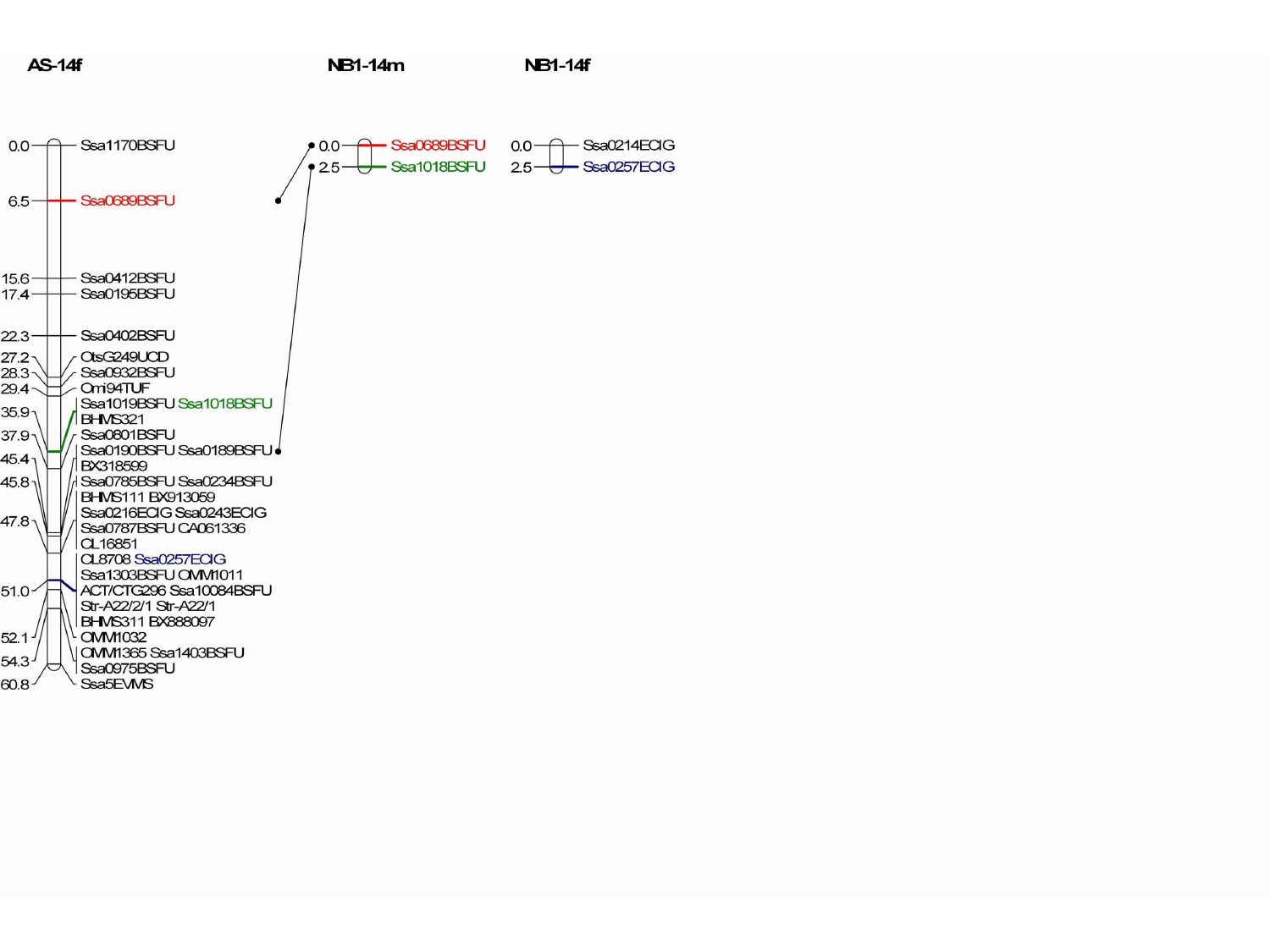

## Slide 7
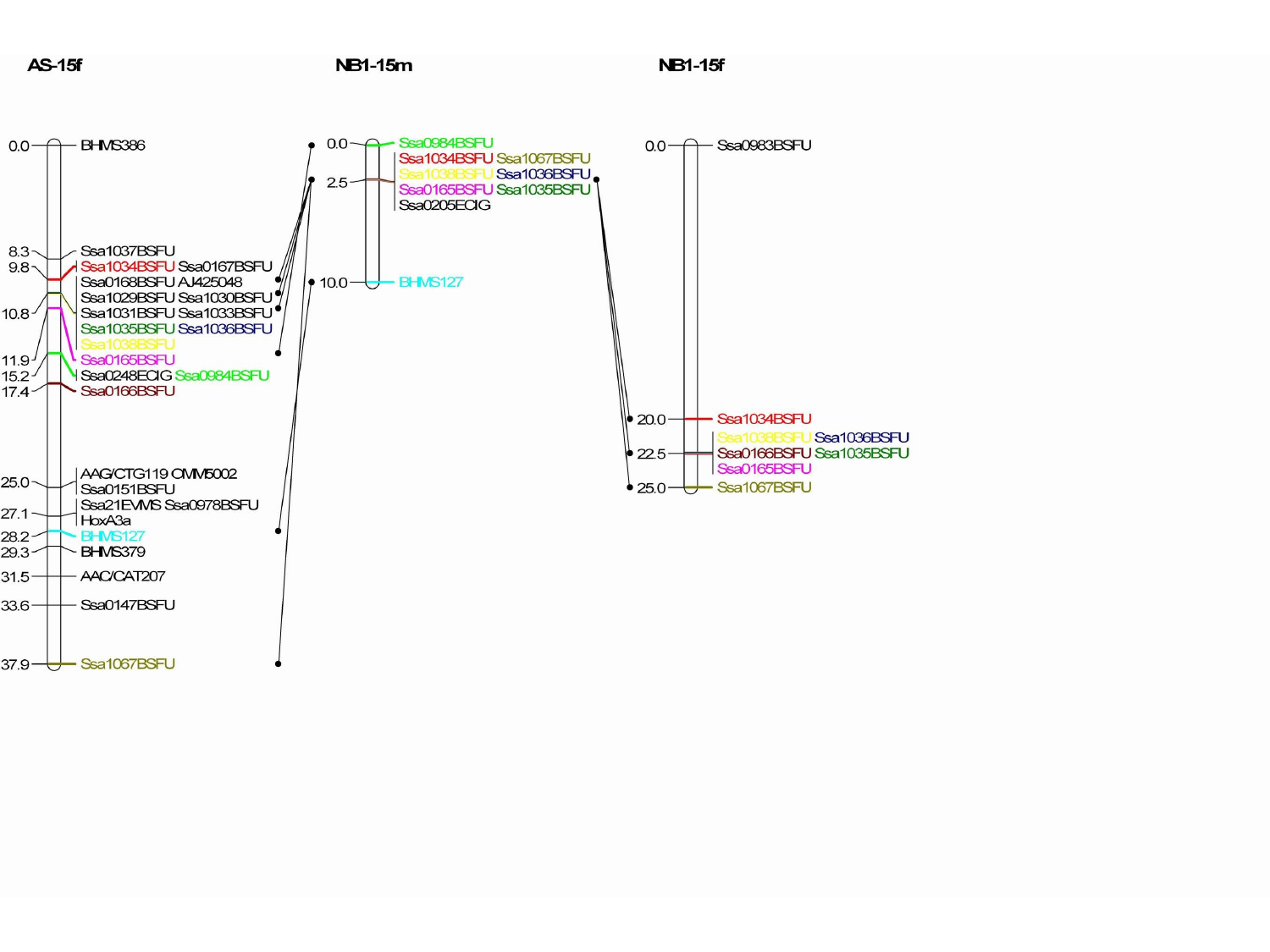

## Slide 8
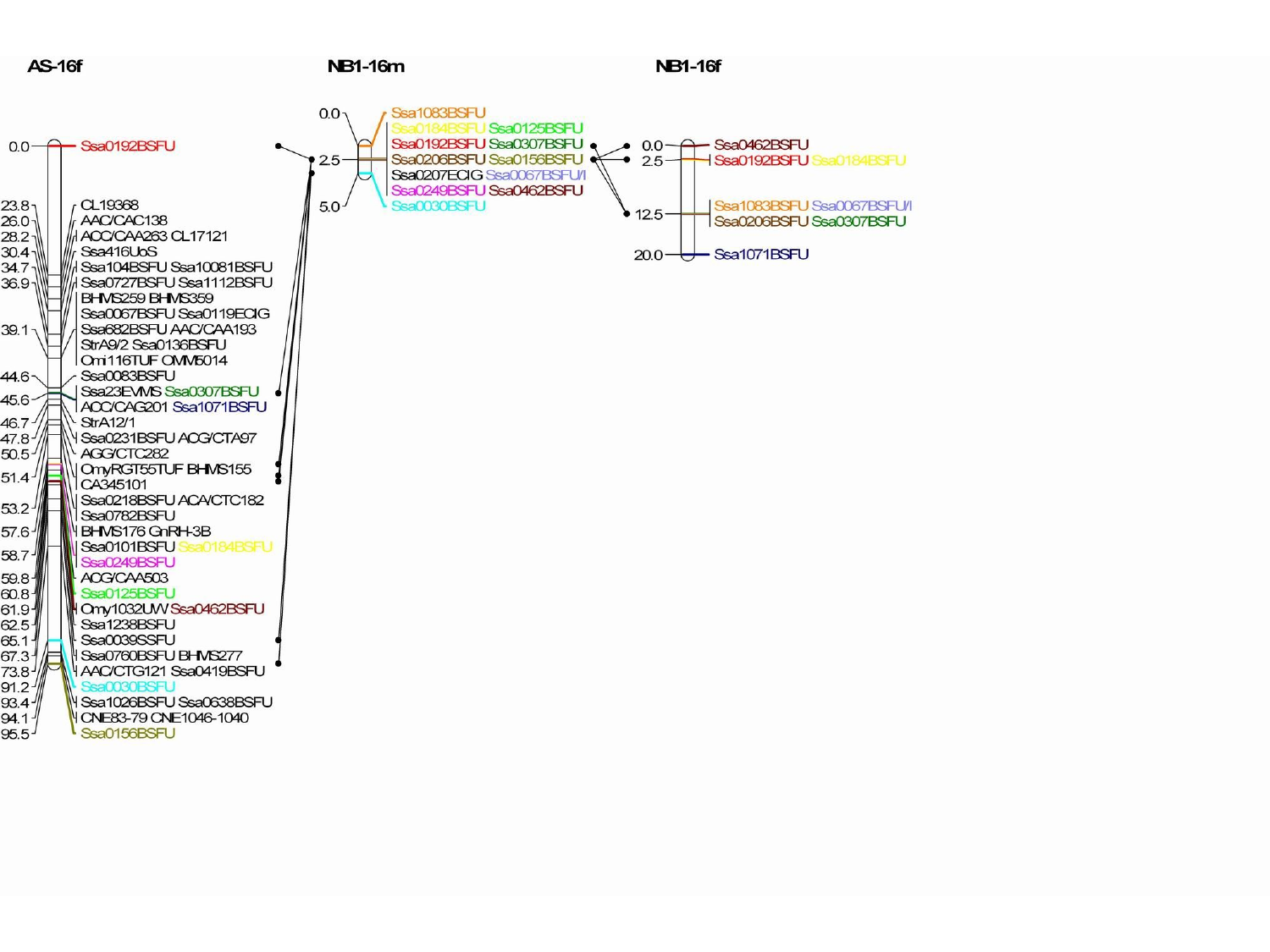

Supplement: Additional file 3 — Figure S2 Continuation of the comparison of the merged SALMAP female linkage groups with the corresponding male-specific and female-specific linkage groups from the NB1 family. [file 1471-2156-11-105-S3.PPTX]

## Slide 1
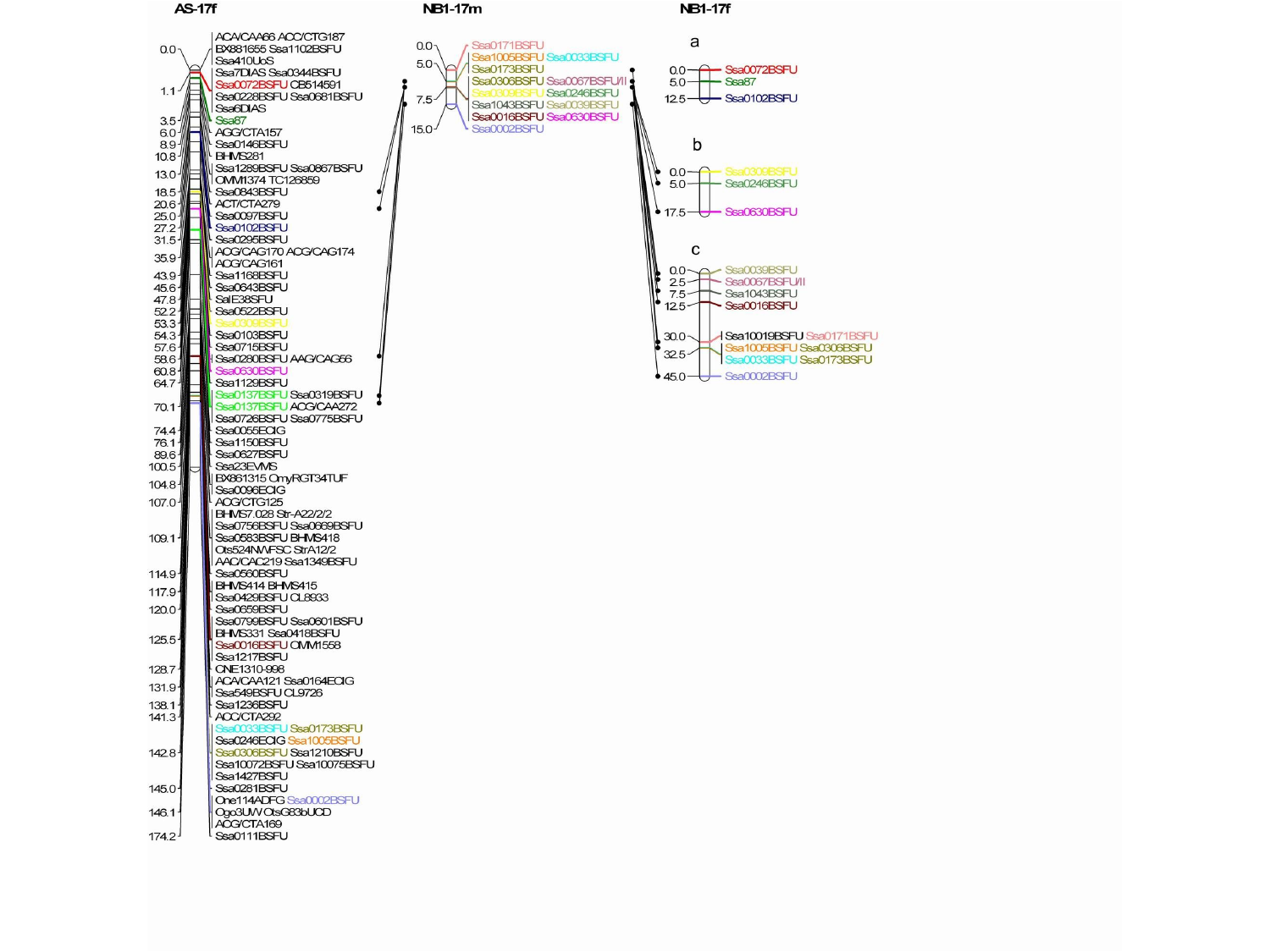

## Slide 2
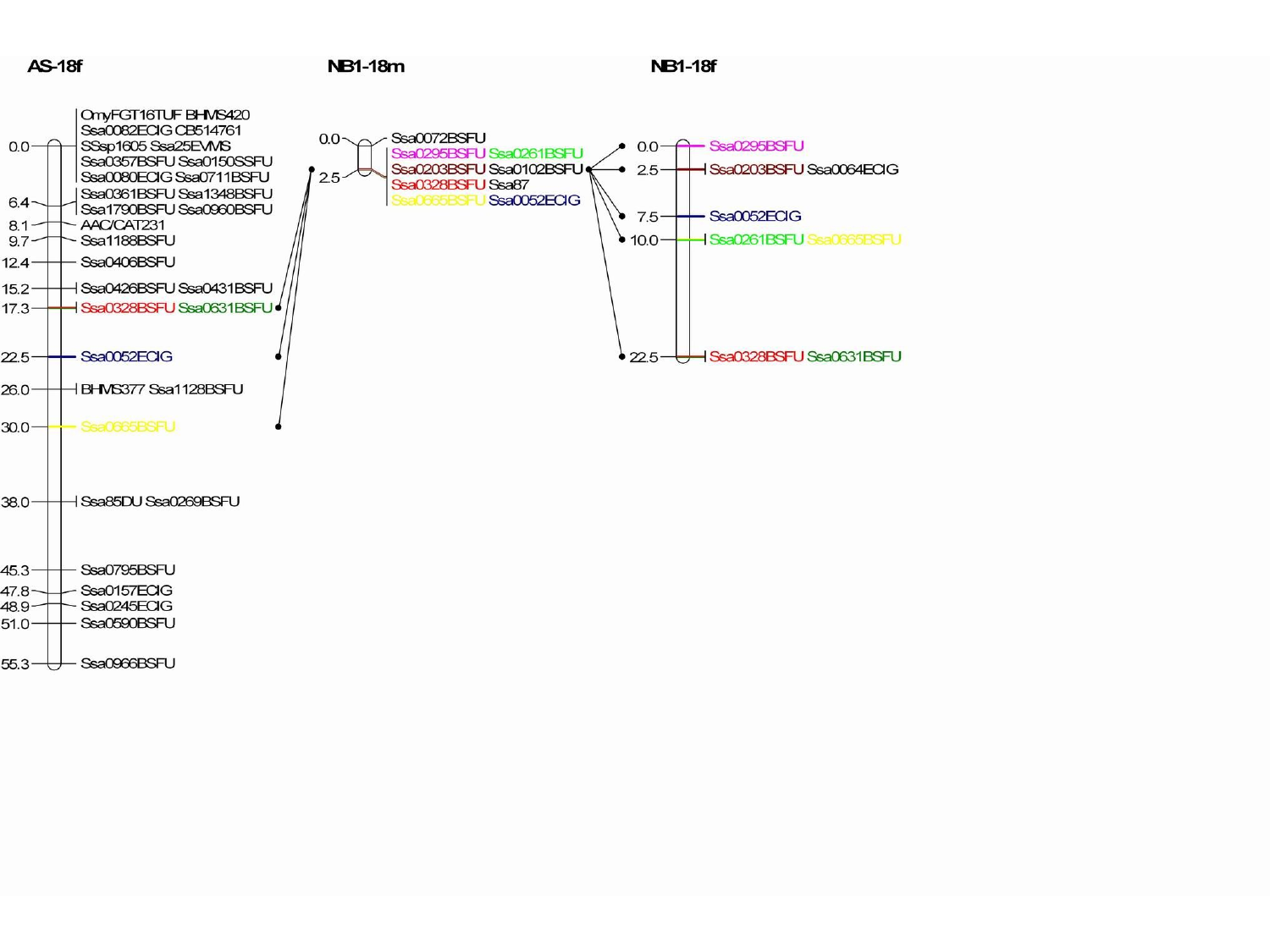

## Slide 3
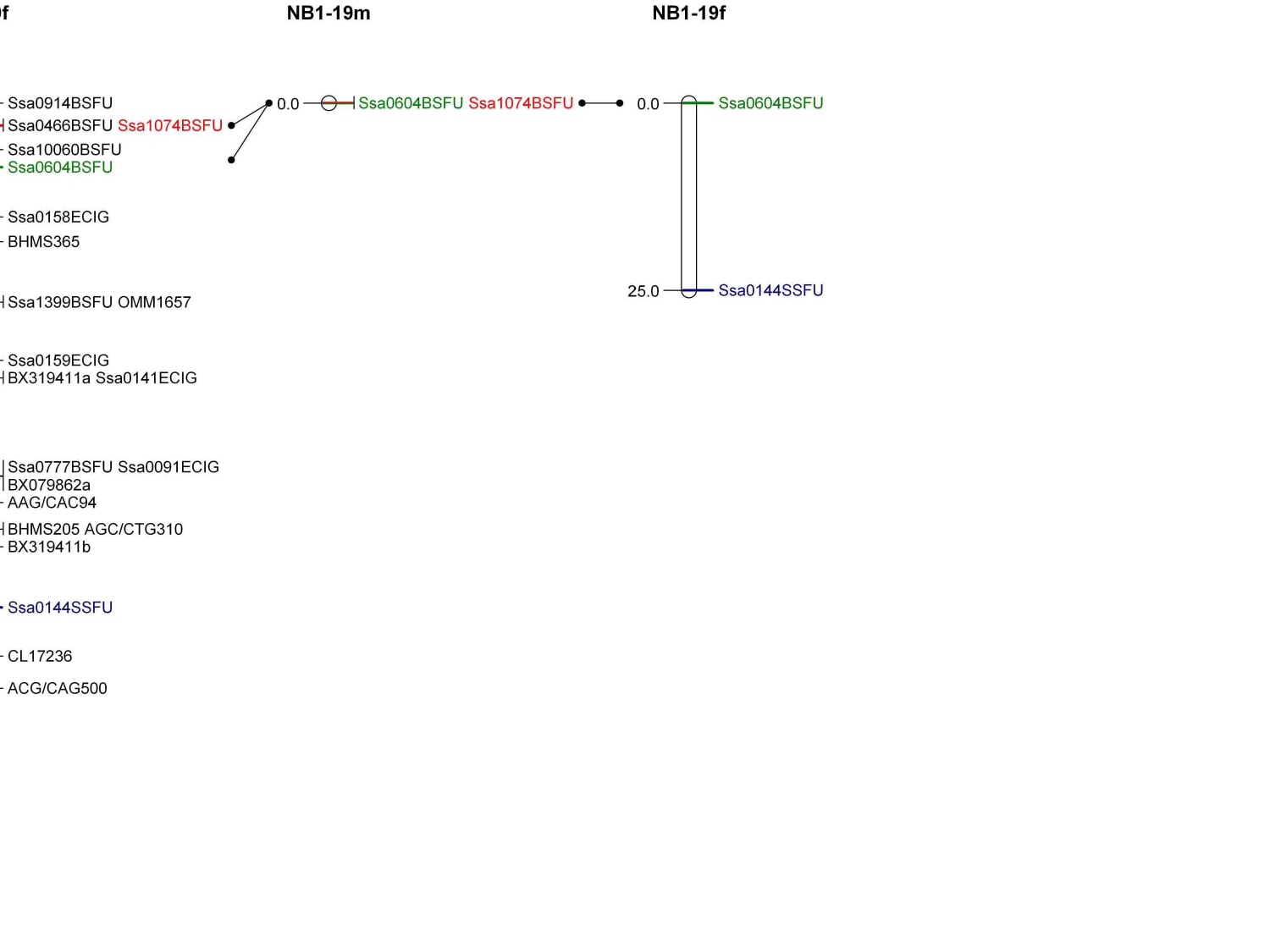

## Slide 4
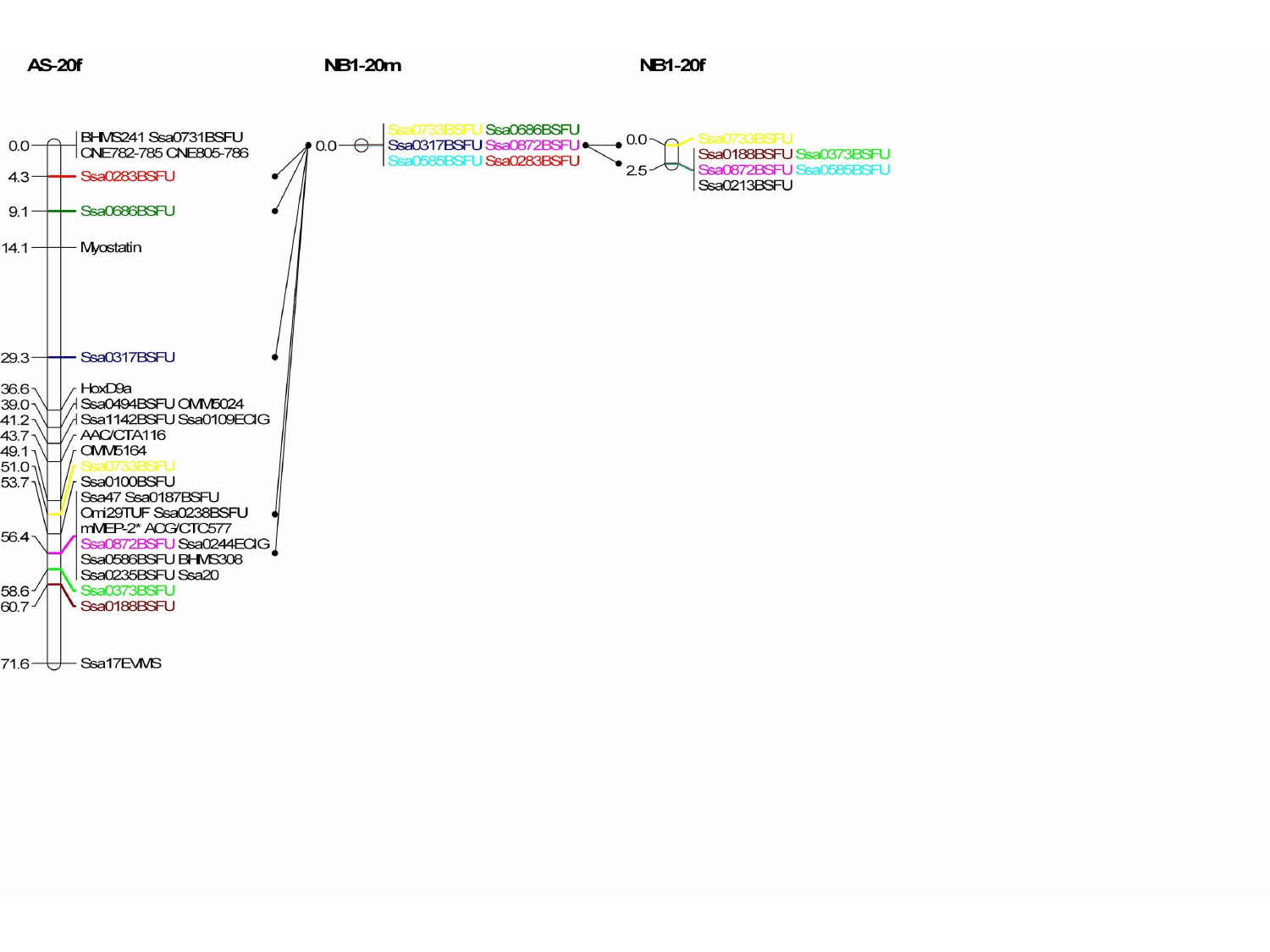

## Slide 5
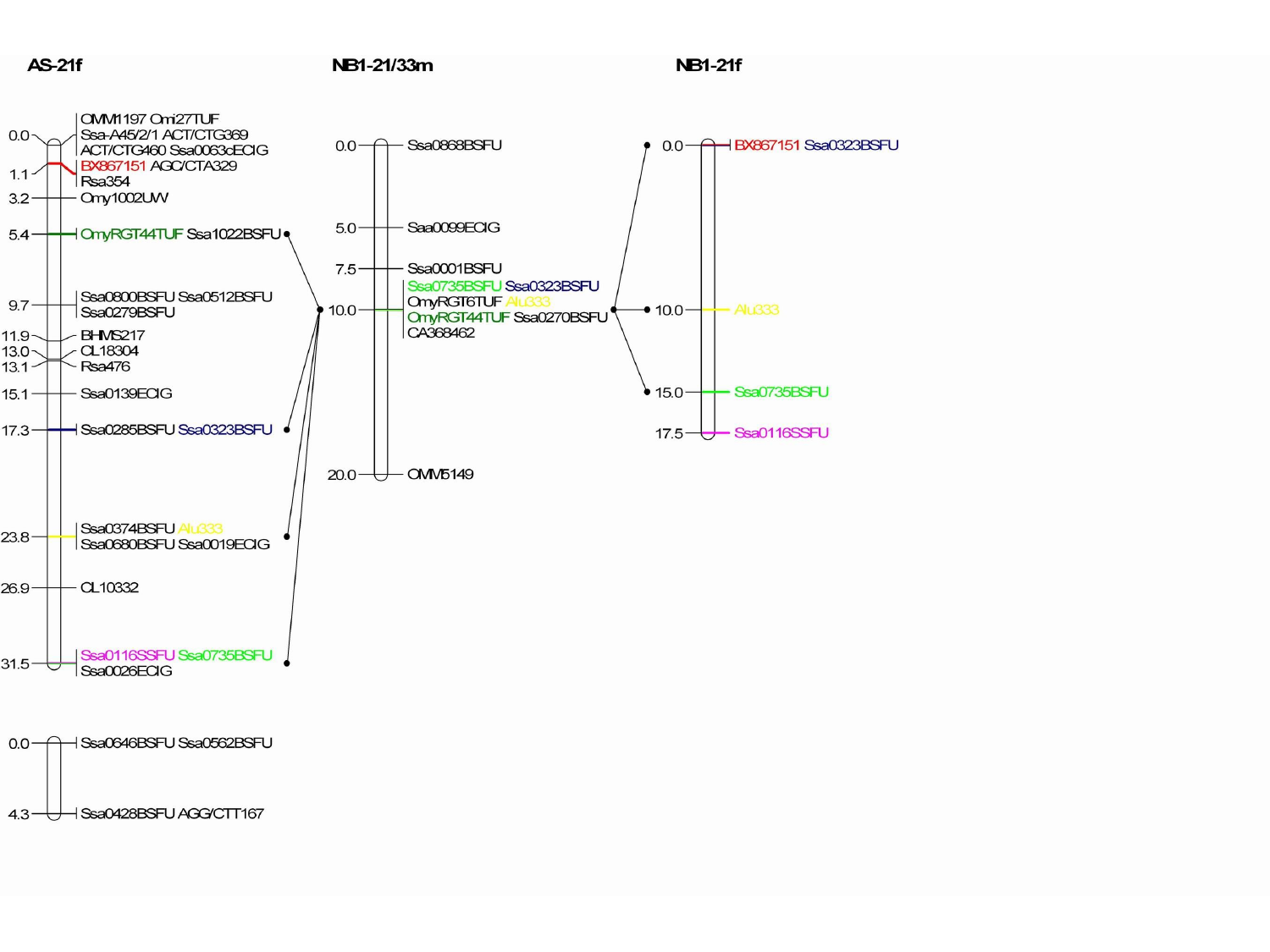

## Slide 6
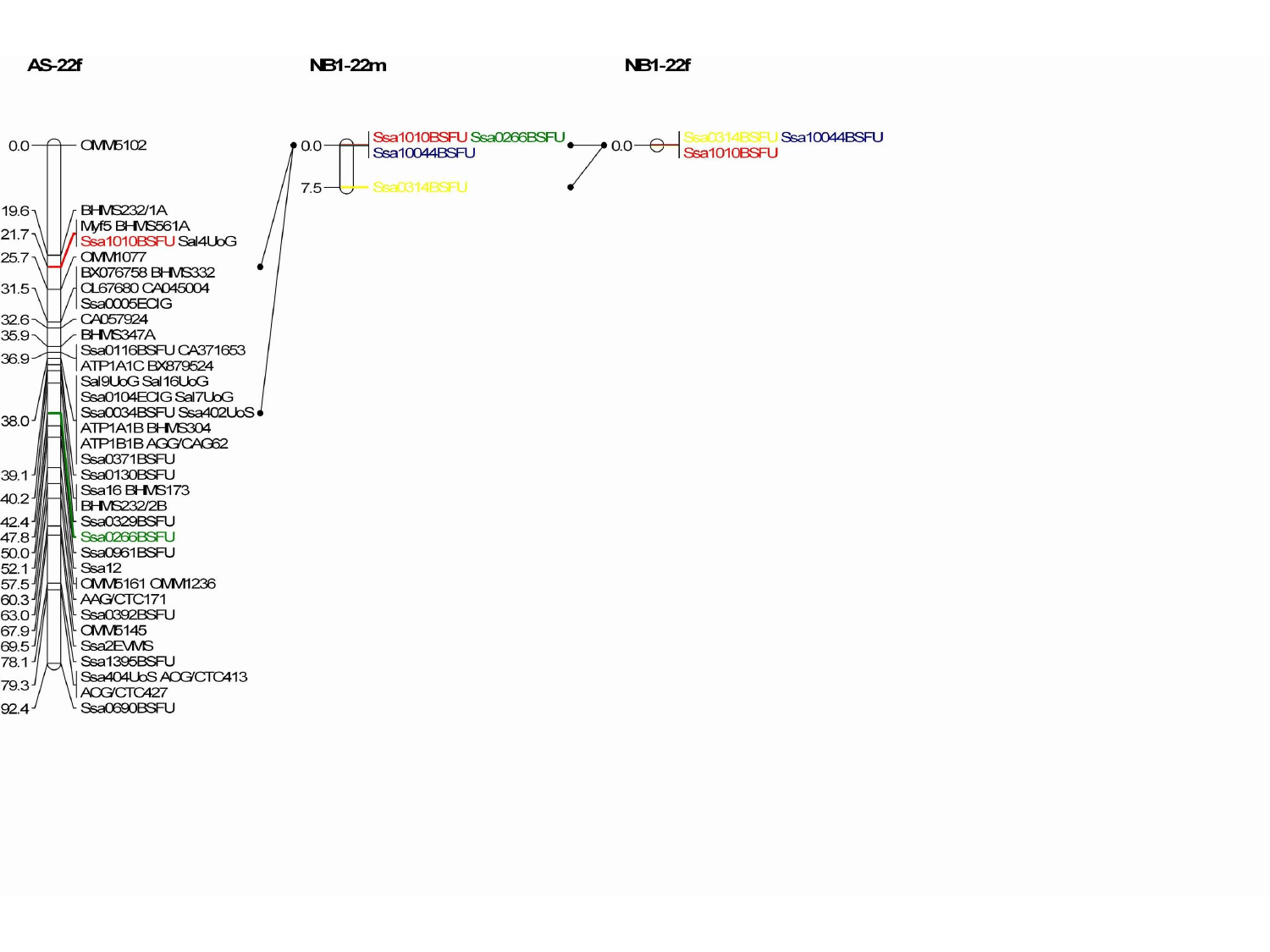

## Slide 7
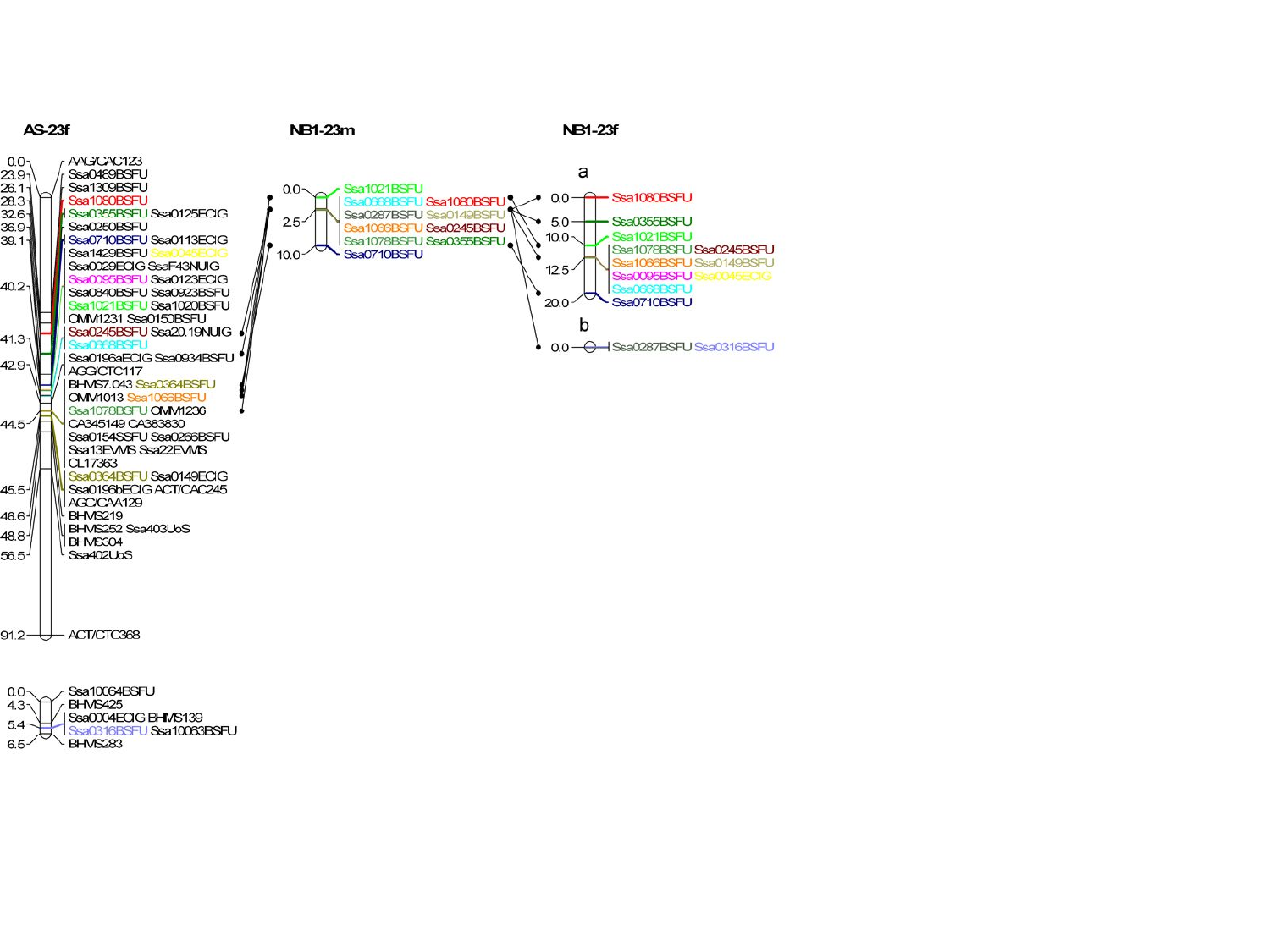

Supplement: Additional file 4 — Figure S3 Continuation of the comparison of the merged SALMAP female linkage groups with the corresponding male-specific and female-specific linkage groups from the NB1 family. [file 1471-2156-11-105-S4.PPT]

## Slide 1
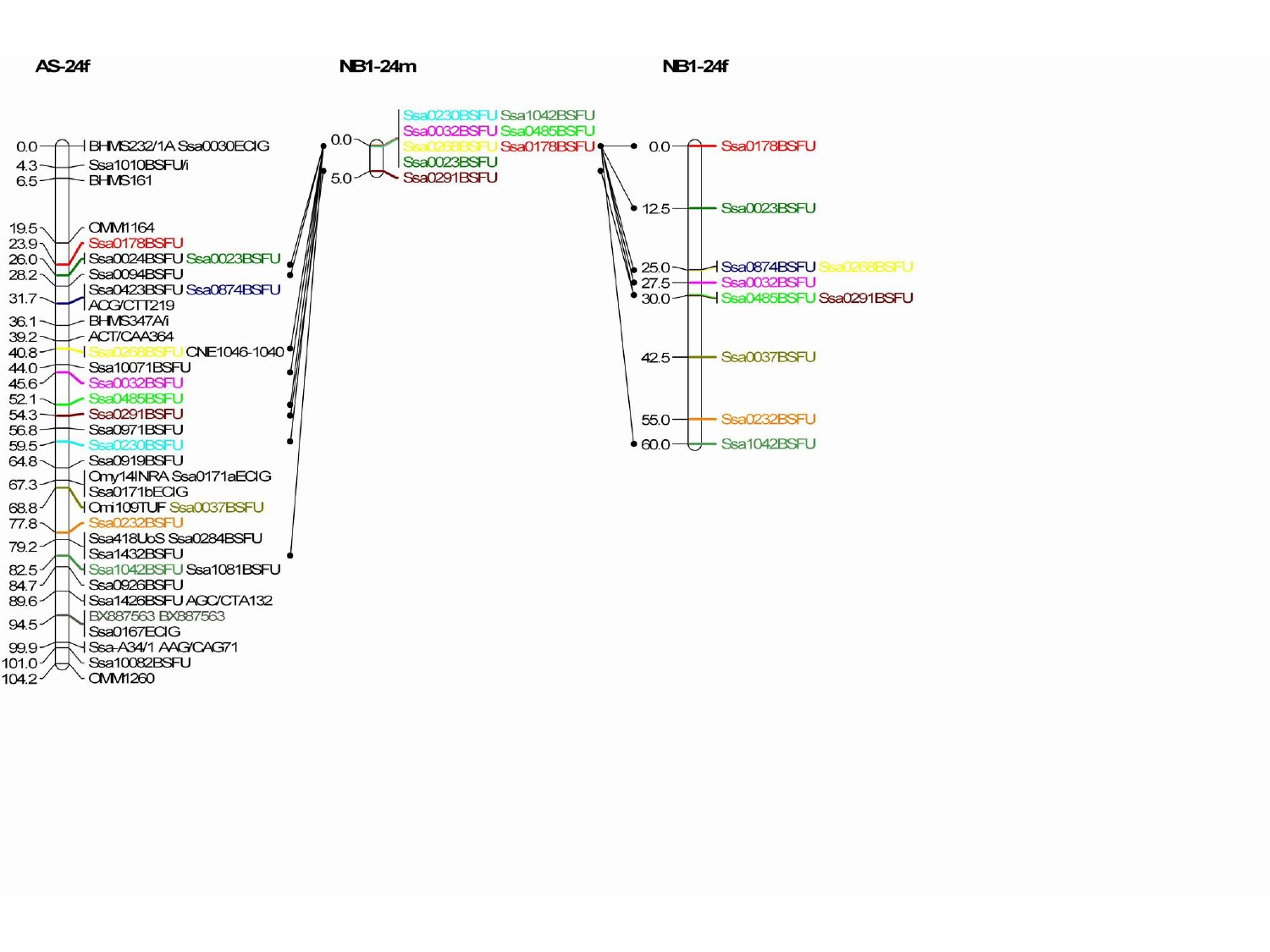

## Slide 2
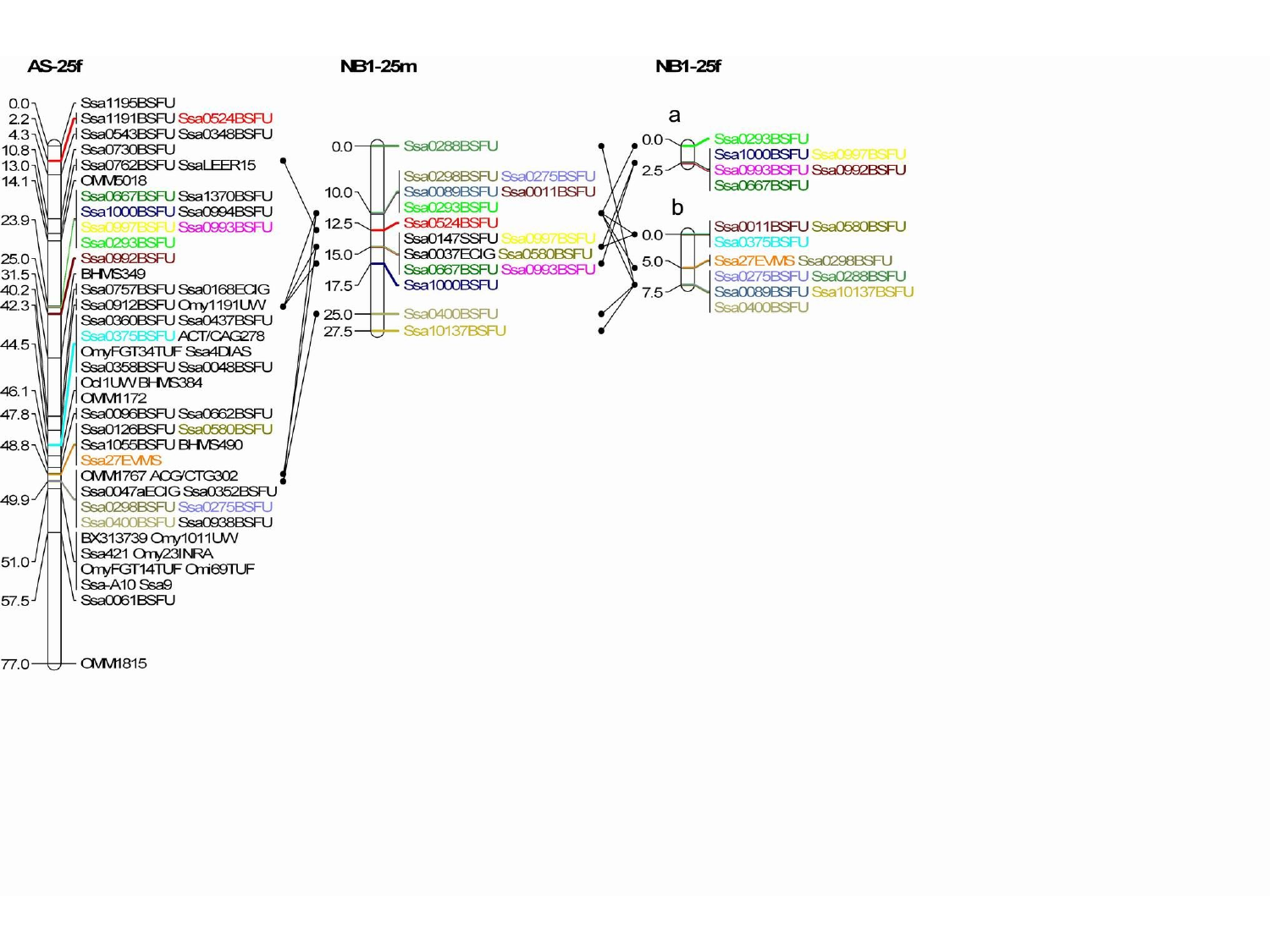

## Slide 3
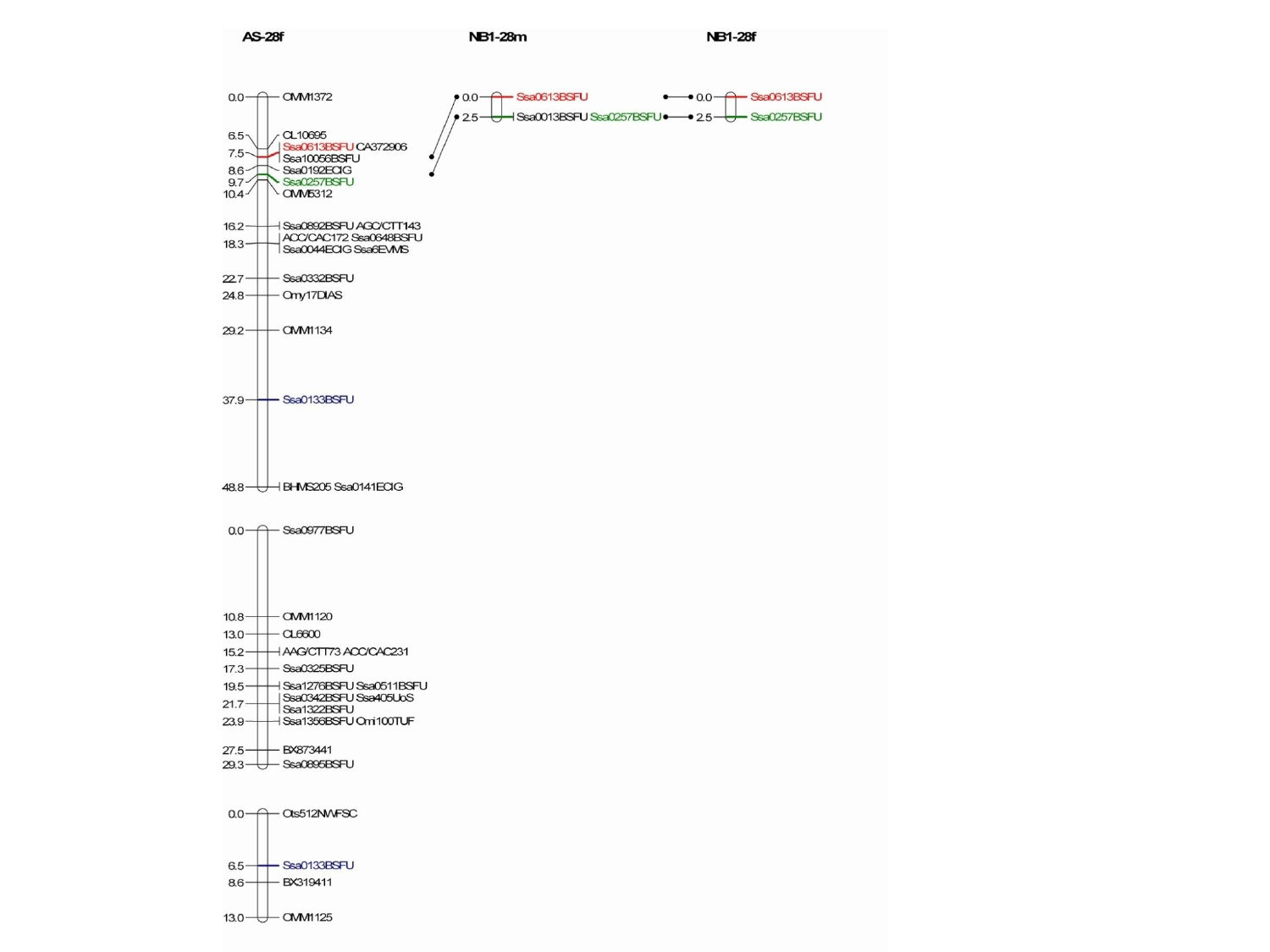

## Slide 4
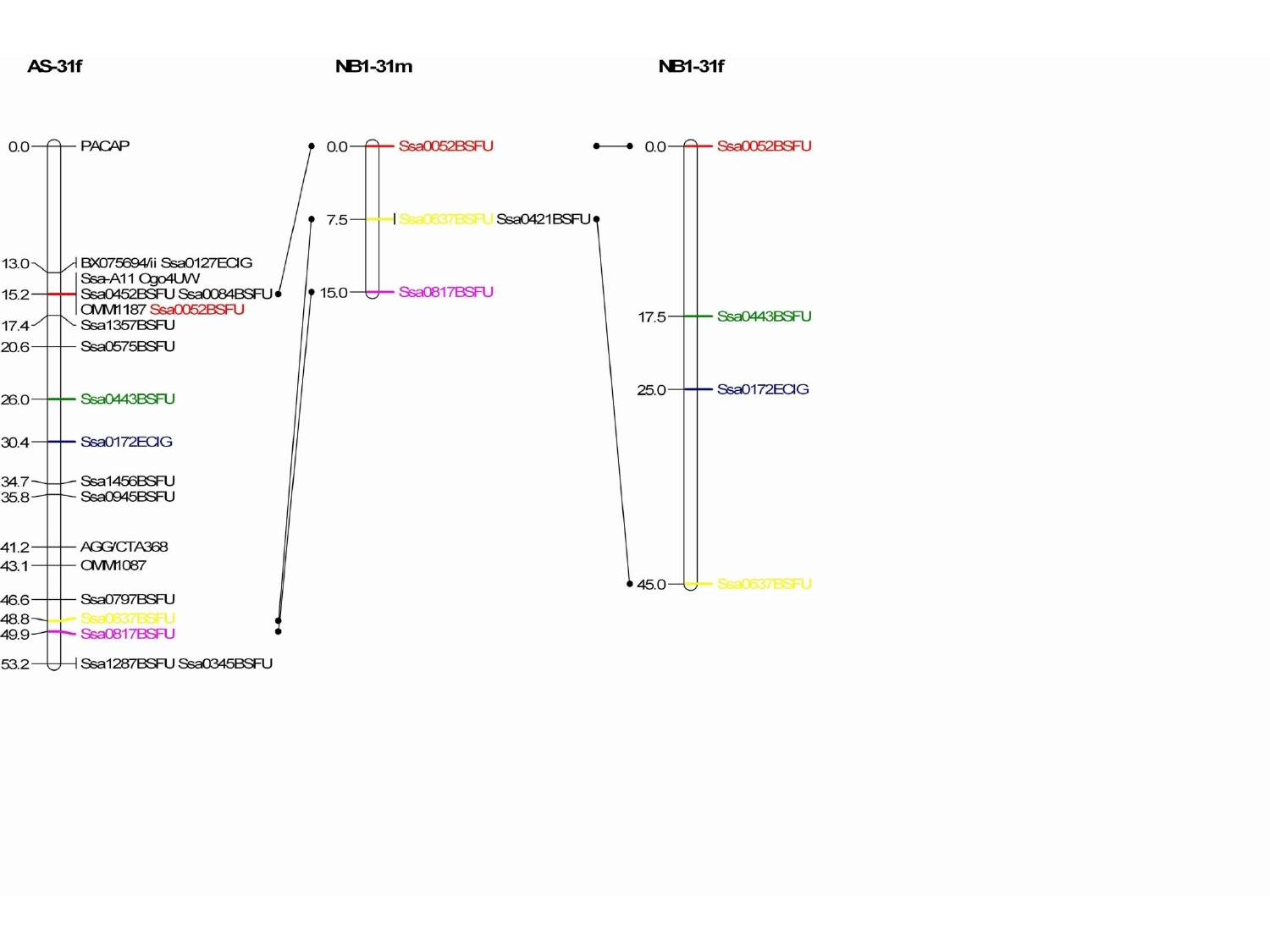

## Slide 5
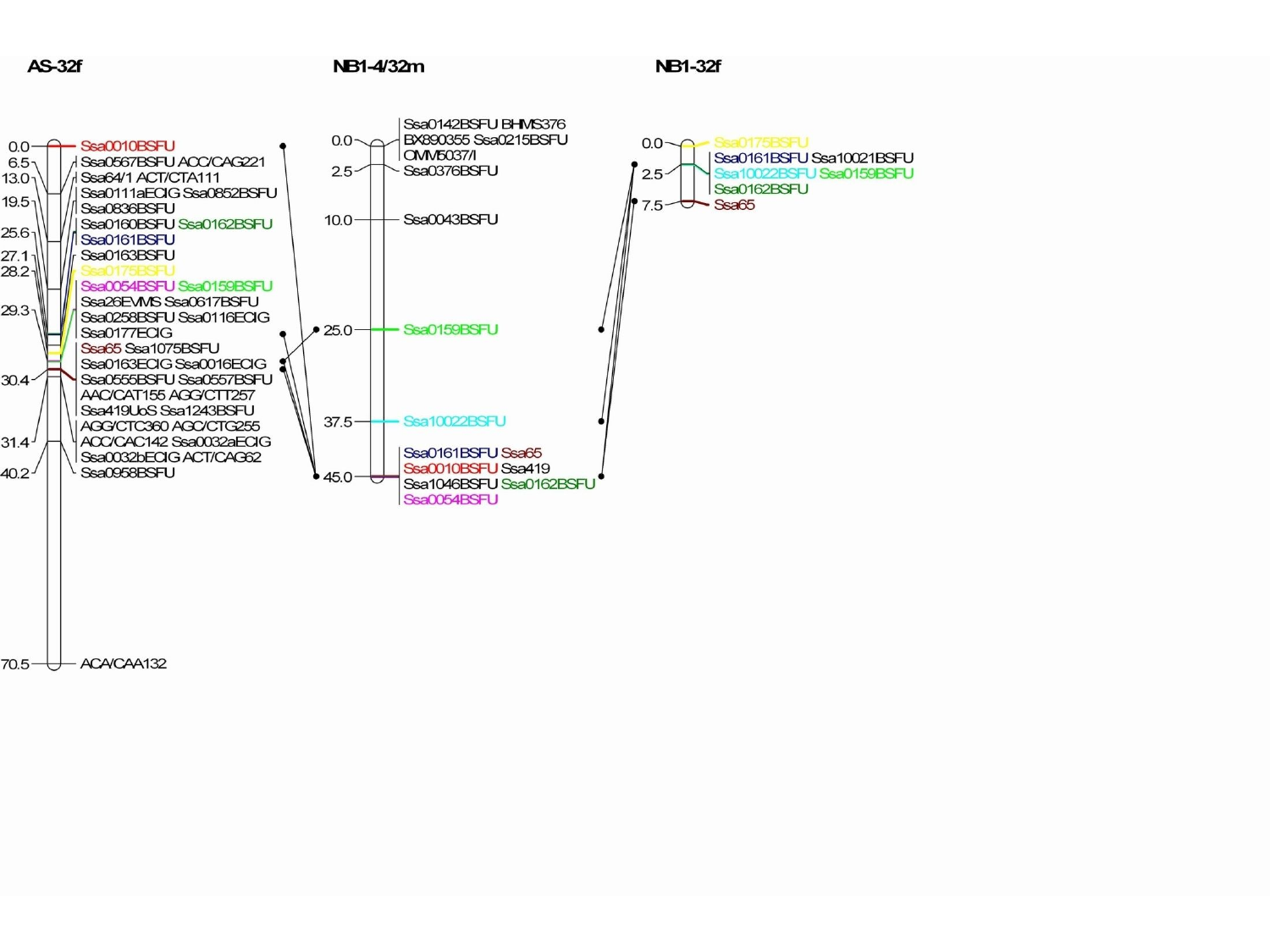

## Slide 6
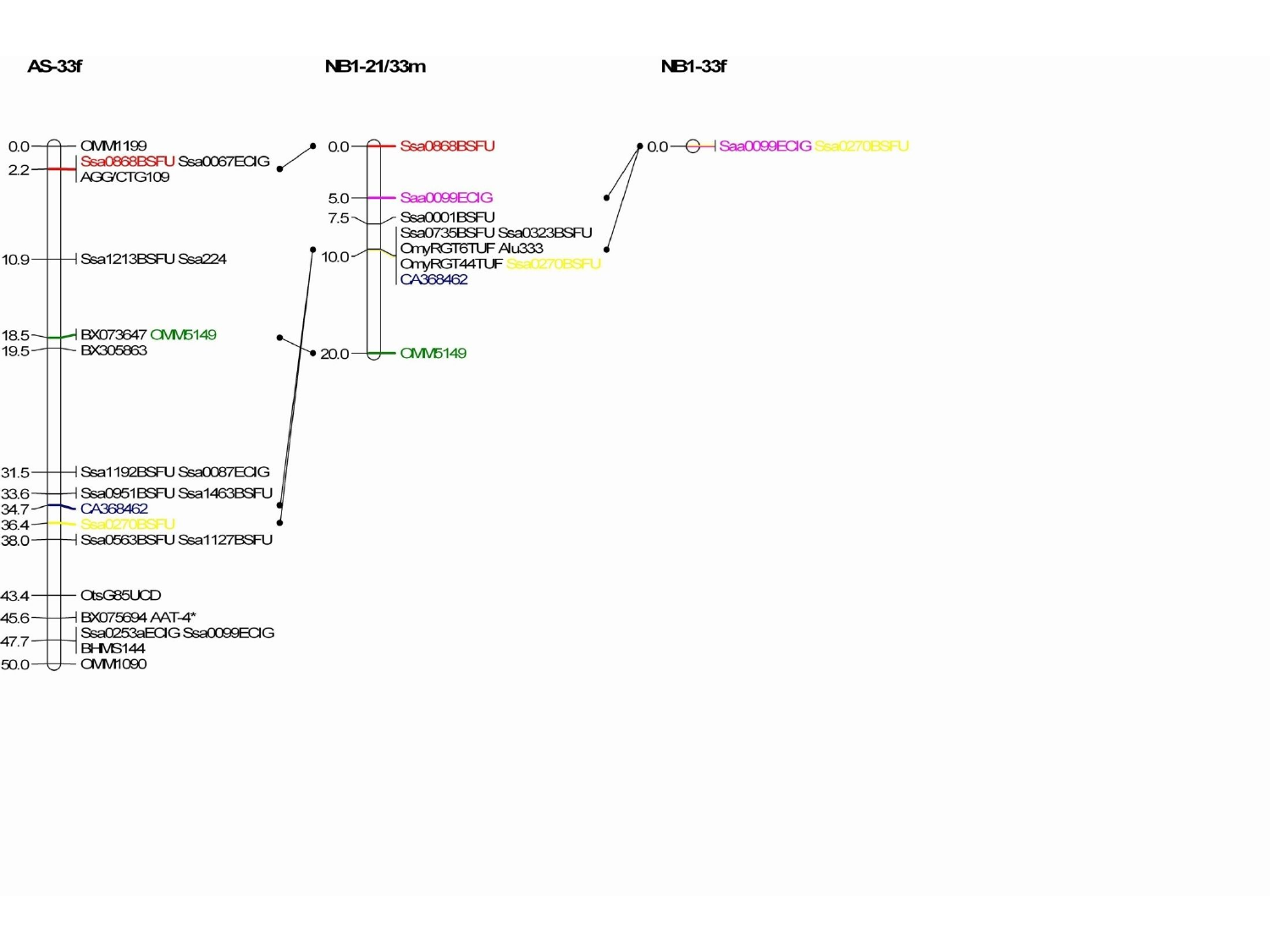

Supplement: Additional file 5 — Figure S4 Continuation of the comparison of the merged SALMAP female linkage groups with the corresponding male-specific and female-specific linkage groups from the NB1 family. [file 1471-2156-11-105-S5.PPTX]
