# Supplementary material for: Comparative genomic analysis of Atlantic salmon, Salmo salar, from Europe and North America
Source: BMC Genet. 2010 Nov 23;11:105. doi: 10.1186/1471-2156-11-105 (PMC2995484; doi:10.1186/1471-2156-11-105)
Supplement: Additional file 6 — Table S2 Phase files for NB1 female-specific linkage groups. [file 1471-2156-11-105-S6.DOCX]

| chrom | **NB1-1af** |
| --- | --- |
| Ssa0124BSFU | **HAHAHAAHHAHHAHHAAHHH-AAHHHHAAAHAAAHAHAHA** |
| Ssa0057BSFU | **HAHAHAAHHAAHAHHAAHHHHAAHHH-AA-HAAAHAHAHA** |
| Ssa1077BSFU | **HAHAHAHHHHAHAHAAAHHHHAAAAHHAAA-AHAHHHAHA** |
| Ssa0063BSFU/II | **HAHAAAHHHHAHAAAHHAHHHAAAAHHAAAHAHHAHHAHA** |
|  |  |
| chrom | **NB1-1bf** |
| Ssa0183BSFU | **HAAHHAAAHAHAAAAAAHAAHAAHAHAHAAHAHAAHHAHA** |
| Ssa0055BSFU | **HAAHHAAAHAHAAAAAAHAAHAAHAHAHAAHAHAAHAAHA** |
| Ssa0233BSFU | **HA-HHAA-HAHAAAAAAHAAHAAHAHAHAAHAHHAHAAHA** |
|  |  |
| chrom | **NB1-2af** |
| Ssa0020BSFU | **AHHHAAHAHHA-AAAAAAHAHHHAAHAAHHHHHH-AHHHH** |
| Ssa1062BSFU | **-H-HAAAAHHAHAAAAAAHAHHHAAHAAHHHHHAAAAHHA** |
| Ssa1006BSFU | **AHHHAHAAHHAHAAAA-AHHHHHAAHAAHHHHHAAAAHHA** |
| Ssa1007BSFU | **AHHHAHAAHHAHAAAAAAHHHHHAA-AAHHHHHAAAAHHA** |
| Ssa0010SSFU | **AHHHAHAHAHAHAAA-AAHHHHHAAAAAHHHHAAAAAHHH** |
| Ssa0844BSFU | **AHHHAHAHAHAA---------HHAAAAAHHHHAAAAAHHH** |
| Ssa0112BSFU | **AHHHAHAHAHAAAAAHAAHHHHHAAAAAHHHHAAAAAHHH** |
| Ssa1086BSFU | **AHHHAHAHAHAAAAAHAAHHHHHA-AAAHHHHAAAAAHHH** |
| Ssa0614BSFU | **-HHHHHAAAHAAAAHHAHHH-HHAAAAAHHHHAAAAAHHH** |
| Ssa0969BSFU | **AHAHAHAHAHHA-AHAHHAHHHHAAAHAHHHHAA-AAHHA** |
|  |  |
| chrom | **NB1-2bf** |
| Ssa0972BSFU | **HHAAAHAAHHAA-AAAAAHHAAAAHAHHAAAHAAAHAAAH** |
| Ssa0290BSFU | **HHAAAH-AHHAAHAAAAAHHAAA-HAHHAAAH-A-HAAAH** |
|  |  |
| chrom | **NB1-3f** |
| Ssa0273BSFU | **AAHAHAHAHAAHHAHHAAHHHAAHHAHAAHAHAAAHAHAH** |
| Ssa0863BSFU | **AAHAHAHAAH-HHHAHAAHHHAAHHAHAAAAHAAAHAHAH** |
| Ssa0377BSFU | **AA---A-AA---H-AHAA--A-AHA--A-AA---AHAHA-** |
| SSsp1606 | **AAHHHAAAAHAAHHAHAAAAAAAHAAHAAAAHAAAHAHAH** |
| Ssa0768BSFU | **AAHHHAAAAHAAHHAHAAAAAAAHAAHAAAAHAAAHAHAH** |
| Ssa0063SSFU | **AAHHHAAAAHAAHHA-AAAAAAAHAAHAAAAHAAAHAHAH** |
| Ssa0153BSFU | **AAHHHAAAAHAAHHAHAAAAAAHHAAHAAAAHAAAHAHAH** |
| Ssa1027BSFU | **AAHHHAAAAHA--HAHAAAAAAHHAAHAAAAHAAAHAHAH** |
| Ssa0518BSFU | **AAHHHAAAAHA--HAHAAAAAAHHAAHAAAAHAAAHAHAH** |
|  |  |
| chrom | **NB1-4f** |
| OMM5037/I | **H-AHAHHAAHAAAHHAAHHH-AAHHHAHAHHAAAHAAHAH** |
| BHMS376 | **HAAHAAHAAHAAAHHAAHHHHAAHHHAHAHHAAAHHAHAH** |
| BX890355 | **HHAHAAHAA-AAAHHAAHHHHAAHHHAHAHHHAAHHAHAH** |
| Ssa0350BSFU | **HHAHAAHHAHAAAHHAAAHAHAAHHHHHAHHHAHHHAHAH** |
| Ssa0058BSFU | **HHHHAAHHAHAHAHHA-AAAHAAHHH-HAHHHAHH-AHAH** |
| Ssa0215BSFU | **AHAHAAHHAAAHAHHHAAAAHAAHAHHHAHHHAHAHAHA-** |
| Ssa0376BSFU | **AAAH-AHHAAAHAHHAHHAA-AAHAA-HAHHHAH-H-HAH** |
| Ssa0789BSFU | **AAAHAAHHAAAH-HHAHHAAHAAHAA-HAHHHHH-HAHHH** |
| Ssa0142BSFU | **AAAHAAHAHAAHHHAAHHAAHAAHAAAHAAHHHHHHAHHH** |
| Ssa0043BSFU | **HAAHAAHHHA-HHH-AHHAAHHHHAAAHAHHHHHHHAHHH** |
|  |  |

| chrom | **NB1-5f** |
| --- | --- |
| Ssa0008BSFU | **-HAAAAHAAAA--AHAAAA--AHHA-A-HHHHHAAAHAAH** |
| Ssa0826BSFU | **HHAAAAHAAAAAAAHHAAAHAAHHAA-AAHHHHAAAHAAH** |
| Ssa0488BSFU | **HHAAAAHAAAAAAAHAAAAHAAHHAAAAAHHHHAAHHAAH** |
| Ssa0338BSFU | **HHAA-AHAAA-AAAHAAAAHAAH-AAAAAHHHHA-AHAA-** |
| Ssa10035BSFU | **HHAAAAHAAAAAAAHAAAAHAAHHAAAAAHHHHAAAHAAH** |
| SSOSL439 | **HHAAAAHAAAAAAAHA-AAH-AHHAAAAAHHHHAAAHAAH** |
| Ssa0240BSFU | **HHAAAAHAAAAAAAH-AAAHHAHHAAAAAHHHH-AAHAAH** |
| Ssa0224BSFU | **HHAAAAHAAAAAAAHAAA-HHAHHAAAAAHHHHAAAHAAH** |
| Ssa0241BSFU | **-HAAAAHAAAAAAAHAAAAHHAHHAAAAAHHHHAAAHAAH** |
| Ssa0941BSFU | **HHAAAA-AAAAA-AHAAAAHHAHHAAA-AHHHHAAAHAAH** |
| Ssa0092BSFU | **HHAAAAHAAAAAAAHAHAAHHAHHAAAAAHHHHAAAHAAH** |
| Ssa0684BSFU | **HHAAAAHAAAAAAAHAHAAHHAHHAA-AA---HAAAHAAH** |
|  |  |
| chrom | **NB1-6f** |
| Ssa0208BSFU | **HHAH-HHAAAAAAAAHAAAAAHAAAAAAHAAAHAHAHHAA** |
| Ssa0484BSFU | **--A-HHHHAAAAAA-H-AA--H-AAA--HA--H-HAHHA-** |
| Ssa0835BSFU | **AHAHHHHHAAAAAAAHAAAAAHAAHAAAHAAAHAHAHHHA** |
| Ssa0825BSFU | **HHAHHHHHAAAA-AAHAAAA-HAAHA-AHAAAHA-AHHHA** |
| Ssa0278BSFU | **HHAHHHHHAAAAAAAHAAAAAHAAHAAAHAAAHAHAHHHA** |
| Ssa0196BSFU | **HHAHHHHHAAAAAAAHAAAAAHAAHAAAHAAAHAHAHHHA** |
| Ssa0554BSFU | **HHAHHHHHAAAAAAAHAAAAAHAAHAAAHAAAHAHAHHHA** |
| Ssa0156ECIG | **HHAHHHHHHAAAAAAHAAAAAHAAHAAAHHAAHAHAHHHH** |
|  |  |
| chrom | **NB1-7f** |
| Ssa0107BSFU | **-HHA-AAHAAHAHAA-HH-AHHAAAHAAAHA-HAHHAHHA** |
| Ssa10023BSFU | **HAHAHHAHAAHAHAAAHHHAAHAAAHAAAHAAHAHHAHAA** |
| Ssa0006BSFU | **HAHA-HAHAHH-HAAAHHH-AHAAA--AAHAAH--HAAAA** |
| Ssa0132BSFU | **HA-AHHAH-HHAHHAAHAHH-HAAAHAA-HAAHHHHAAAA** |
| Ssa0937BSFU | **HAHAHHAHAHHAHHHHHAHH-HAAHH-AAHAAA-HHAAAA** |
|  |  |
| chrom | **NB1-8f** |
| Ssa0957BSFU | **-HAAA-A-AHAAHHAAHHAAAHHAHAH-AHAAHHHAHHHH** |
| Ssa0051BSFU | **HHAAAAAAAHAAHHAAHHAAAHHAHAHHAHAAHHHAHHHH** |
| Ssa0821BSFU | **HHAAAAAAAHAAHHAAHHAAAHHAHAHHAHAAHHHAHHHA** |
| Ssa0918BSFU | **HHHAAAAAAHAHHHAAHHAAAHHAHAHHAHAAHHHAAHHA** |
| Ssa0227BSFU | **HHHAAAAAAHAAHHAAHHAAAHHAHAHHAHAAHHHAAHHA** |
| Ssa0226BSFU | **HHHAAAAAAHAAHHAAHHAAAHH-HAHHAHAAHHHAAHHA** |
| Ssa0050BSFU | **HHHAAAAAAHA-HHAAHHAAA-HAHAH-AHAAHHHAAHH-** |
| Ssa0218ECIG | **-HHA-A----A-HHA----A-H--H-HA-H-A-H--AHAA** |
| Ssa0666BSFU | **HHHAAAAAAHAHHHAHHHAAAHHAHAHAAHAAHHHAAHAA** |
| Ssa197DU | **HHHAAAAAAHAHHHAHAHAAAHHAHAHAAHAAAHHAA-AA** |
|  |  |

| chrom | **NB1-9f** |
| --- | --- |
| Ssa0229BSFU | **AHAAAAHAHHHHAAHHHAAHAHHHAAHAHA-AAAHAAAHA** |
| Ssa0503BSFU | **-HAAAAH-HHHH-AHHHAAH-HHHAA-AHAHAAA-AAAHA** |
| Ssa1052BSFU | **AHAAAAHHHHHAAAHHHAAAAHHHAAHAHAHAAAHAAAHA** |
| Ssa0091BSFU | **AHAAAAHHHHHAAAHHHHH-AHHHAAHAHAHAAAHAAAAA** |
| SSsp3016 | **-HHAHA-HHHHAAA-HH-HAAAHHA-AHHAHAH-HAAAAA** |
| Ssa0028ECIG | **----HA-H--HA---HHHHA-AH--A-H-A-AHAH--A-A** |
| Ssa0122BSFU | **AHHAHAHHHHH-AA-HHH-AAAHHAAAHHAHAHAHAAAAA** |
| Ssa0819BSFU | **HAHAHAHHHHHAAAHHHHHAAAHHAAAHAAHAHAHAAAAA** |
| Ssa0254BSFU | **HAHAHAHHHHHAAAHHHHHAAAHHAAAHAAHAHAHAAAAA** |
| Ssa0592BSFU | **HAHAHAHHHHHAAAHHHHHAAAHHAAAHAAHAHAHAAAAA** |
| Ssa1016BSFU | **HA-AHAHHHHHAAAHHHHHAAAHH-AAHAAHAHAHAHAAA** |
|  |  |
| chrom | **NB1-10af** |
| Ssa0042BSFU | **HHHAHHAAHAAHHHHAHAHAHAAAAAAAHAAHHAAHAHHH** |
| Ssa1070BSFU | **HAAAAAAAAAAHHHHA-AHHHAAAAAAAHAAHHAAHAHHH** |
|  |  |
| chrom | **NB1-10bf** |
| Ssa0420BSFU | **HAAHHHAAAAAHHAHAHHAHHAAAHAAAAHHHAHAAHHAH** |
| Ssa0769BSFU | **H-AHHHAHAAAHHAHAAHAHAAHHHAAAAHHHAHAAHHAH** |
| Ssa0885BSFU | **HAAHH-AHAAAHHAAAAHAHAAHHHAAAAHHHAHAAHHAH** |
| Ssa0038BSFU | **HAAHHHAH-AAH-AAAAHAHAAHHHAHAHHHAAHHAHHHH** |
| Ssa0850BSFU | **HAAHHHAHAAAHHAAAAHAHAAHHHAHAHHHAAHHAHHHH** |
| SSOSL85 | **H-HHH-HHAAAHHAAAAHHHAAHAHAHAHHHAAHHAAHH-** |
| Ssa0815BSFU | **HAHHHHHHA-AAAA-AAHHH-AHAHAHAHHHAAHHHAHHH** |
|  |  |
| chrom | **NB1-11f** |
| Ssa0670BSFU | **HHAAAAHAAAHAHAAAHAAHAHHHHHAAHAHHAAHAHHHH** |
| SsaD144 | **HHAAAAAAAAHAHAAAHAAHAHHHHHAAHAHHHAHAHHHH** |
|  |  |
| chrom | **NB1-12af** |
| Ssa0808BSFU | **HAAAHHAAAHHA-HAHHHAAHHAAAAAAAAHAAAHAAHAH** |
| Ssa0571BSFU | **HAAAHHAAAHHAAHAHHHAAHAAAHAAAAHHAAAHHAHAH** |
| Ssa0256BSFU | **HAAAHHHAAHHAAAAHAHAAHAHAHAAAAHHAAAHHHHAH** |
|  |  |
| chrom | **NB1-12bf** |
| Ssa0747BSFU | **-HHHAAA-HAAAHHHAHAHHA-HHAAHAAAAAHHAAHHHH** |
| Ssa0962BSFU | **AHHHAAAHHAAAHHHAHAHHAAHHAAHAAAAAHHAAHAHH** |
|  |  |
| chrom | **NB1-12cf** |
| Ssa0739BSFU | **AAAHHHHAAAAHAAAAAHAAAAHHHAAAAHHAAHAHHAAA** |
| Ssa0897BSFU | **AAAHHHHAAAAHAAAAAHAAAAHHAAAAHHAAAH-HHAAA** |
|  |  |
| chrom | **NB1-13f** |
| Ssa0138BSFU | **HHHHHAAAAAH-HHAHAA-HAAHAHAAAAAAHHAHAAAHA** |
| Ssa1041BSFU | **HHHHHAAAAA-AHHAHAAAHAAHA-AAAAAAHHAHAAAHA** |
| Ssa0853BSFU | **HHHHHAAAAAHA-HAHAAAHAAHAHAAAAAAHHAHAAAHA** |
| Ssa164 | **HHHH-AAAAAAAHHAHAAAHAAHAHAAAAAAAHHHAAAHA** |
| Ssa0858BSFU | **HHHHHAAAAAAAHHAHAAHAAAHAH-A-AAAAHHHAAAHA** |
|  |  |
| chrom | **NB1-14f** |
| Ssa0214ECIG | **AAAHAHHHAAHAHHAAHHHHAHAAHAAAA-AHAHHAAA-A** |
| Ssa0257ECIG | **AAAHAHHHAAHAHHAAHHHHAHAAHAAAAAAHAHHAHAHA** |
|  |  |
| chrom | **NB1-15f** |
| Ssa0983BSFU | **AHAAHHHHA-HAAHHAAHAAHAAAHA-AAH-AAHHAAHAA** |
| Ssa1034BSFU | **AHAAHAHHAHHA-HAAHHAHHAAAHAAAAHAHHHHHAHAA** |
| Ssa1038BSFU | **AHAAHAHHAHHAHHAAHHAHHHAAHAAAAHAHHHHHAHAA** |
| Ssa1036BSFU | **AHAAHAHHAHHAHHAAHHAHHHAAHAAAAHAHHHHHAHAA** |
| Ssa0166BSFU | **AHAAHAHHAHHAHHAAHHAHHHAAHA-AAHAHHHHHAHAA** |
| Ssa1035BSFU | **AHAAHAHHAHHAHHAAHHAHHHAAHAAAAHAHHHHHAHAA** |
| Ssa0165BSFU | **AHAAHAHHAHHAHHAAHHAHHHAAHAAAAHAHHHHHAHAA** |
| Ssa1067BSFU | **AHAAHAHH-AHAHHAAHHAHHHAA-AAAAHAHHHHHAHAA** |
|  |  |
| chrom | **NB1-16f** |
| Ssa0462BSFU | **-AAHHAHHHHHA-AAAHAHA-HHAHAAAHHHHAA-HAAAH** |
| Ssa0192BSFU | **HAAHHAHHHHHAHAAAHAHAAHHAHAAAHHHHHAHHAAAH** |
| Ssa0184BSFU | **HAAHHAHHHHHAHAAAHAHAAHHAHAAAHHHHHAHHAAAH** |
| Ssa1083BSFU | **HAAHHAHAHHAAHAAAHAHAAHH-HAHAAHHHHAHHAAAH** |
| Ssa0067BSFU/I | **HAAH-AHAHHAAHAAAHAHAAHHAHAHAAHHHHAHHAAAH** |
| Ssa0206BSFU | **HAAHHAHAHHAAHAAAHAHAAHHAHAHAAHHHHAHHAAAH** |
| Ssa0307BSFU | **HAAHHAHAHH--HAA---H-AHHAHAHAAHHHHAHH---H** |
| Ssa1071BSFU | **AAAHHAHAHHAHHAAAHAHAAH-A-AHAAHHHAAHHAAA-** |
|  |  |
| chrom | **NB1-17af** |
| Ssa0072BSFU | **HHAHAAHAHHAHAHAAAHAHHAHHHHAHAAHAAAAAHHHA** |
| Ssa87 | **HHAHAAHAHHAHAHAAAHAHHAHAHHAAAAHAAAAAHHHA** |
| Ssa0102BSFU | **AHAHA-HAHAAHAHAAAHAHHAHAHH-AA-HAAAAAHAHA** |
|  |  |
| chrom | **NB1-17bf** |
| Ssa0309BSFU | **---A--HAH-HH-AA-HAAHHHHAAAAHAAAHAAAHHHHA** |
| Ssa0246BSFU | **AAHAHHHAHHHHAAAAHAA-HHAAAAHHAAAHAAA--HHA** |
| Ssa0630BSFU | **AAHAAHHAHAHHAAAAHAHHHHAAAAHHAHAHAAAHHHAA** |
|  |  |
| chrom | **NB1-17cf** |
| Ssa0039BSFU | **-AHAAHHAHAHAAAAHHHHAHHAAAHHHHH-AAAAAAAAA** |
| Ssa0067BSFU/II | **HAHA-HHHHAHAAAAHHHHAHHAAAHHHHHAAAAAAAAAA** |
| Ssa1043BSFU | **HAHAAAHHHAHAAAHHHHHAHHAAAHHHHHAAAAAAAAAA** |
| Ssa0016BSFU | **AAHAAAHHHAHAAAHHHHHAHHAAHHHHHHAAAAAAAAAA** |
| Ssa10019BSFU | **HAHAAAHHAAHAAAHH--HAHAAHHHHHHHAAHAAAAAAA** |
| Ssa0171BSFU | **HAHAAAHHAAHAAAHHAAHA-AAHHHHH--AAHA-AAAAA** |
| Ssa1005BSFU | **HAHAAAHHAAHAAAHHAAHAHAAHHHHHHAAAHAAAAAAA** |
| Ssa0306BSFU | **HAHAAAHHAAHAAAHHAAHAHAAHHHHHHAAAHAAAAAAA** |
| Ssa0033BSFU | **HAHAA-HHAAHAAAHHAAHAHAAHHHHHHAAAHAAAAAAA** |
| Ssa0173BSFU | **HAHAAAHHAAHAAAHHAAHAHAAHHHHHHAAAHAAAAAAA** |
| Ssa0002BSFU | **HHA--AHHAAHAAAHHAAHAHAA-HHHHHAAAHA--HHHA** |
|  |  |
| chrom | **NB1-18f** |
| Ssa0295BSFU | **AH-HAH---AAHAAAAHHHHAAA-AHAAAHHAAAAA---A** |
| Ssa0203BSFU | **AHAAAHHAAAAHAAAAHHHHAAAAAHAAAHHAAAAAHAHA** |
| Ssa0064ECIG | **AHAAAHHAAAAH-AAAHHHHAAAAAHAAAHHAAAAAHAHA** |
| Ssa0052ECIG | **AH-AAHHA----AAAA-AHHA---A-A-A-HAAA--A-HA** |
| Ssa0261BSFU | **AHAAAHHAAAHHAAAAHAHHAAAAAHAAAHHAAAAAAAHA** |
| Ssa0665BSFU | **AHAAAHHAAAHHAAAAHAHHAA-AAHAAAHHAAAAAAAHA** |
| Ssa0328BSFU | **A-AAAA-A-HHHAHAAHA--AAAHAHAAAH-AAAAAAAHA** |
| Ssa0631BSFU | **AHAAAAHAHHHHAHAAHAHHAAAHAHAAAHHAAAAAAAHA** |
|  |  |

| chrom | **NB1-20f** |
| --- | --- |
| Ssa0733BSFU | **AHAHAAAAAAAHAAHAAAAHHAAAHAAHAAAHHAAAHAHH** |
| Ssa0188BSFU | **AHAHAAAHAAAHAAHAAAAHHAAAHAAHAAA-HAAAHAHH** |
| Ssa0373BSFU | **AHAHAAAHAAAHAAHAAAAHHAAAHAAHAAA-HAA-HAHH** |
| Ssa0872BSFU | **AHAHAAAHAAAHAAHAAAAHHAAAHAAHAAAHHAAAHAHH** |
| Ssa0585BSFU | **AHAHAAAHAAA-AAHAAAAHHAAAHAAHAAAHHAAAHAHH** |
| Ssa0213BSFU | **AHAHAAAHAAAHAAHAAAAHHAAAHAAHAAAHHAAAHA-H** |
|  |  |
| chrom | **NB1-21f** |
| BX867151 | **HHHAAHAAAAAAHHHAHAAAHAHAHAAAHAAAAAHHHHAA** |
| Ssa0323BSFU | **HHHAAHAAAAAAHHHAHAAAHAHAHAAAHAAAAAHHHHAA** |
| Alu333 | **HAHAAHHAAAAAH-HAHAAAHAHAHAHAAAAAAAHHHHAA** |
| Ssa0735BSFU | **HAHAAHHAAAAAHHHAHAAAHAHAHHHAAAAHAAHHHHAA** |
| Ssa0116SSFU | **HAHAAHHHAAAAHHH-HAAAHAHAHHHAAAAHAAHHHHAA** |
|  |  |
| chrom | **NB1-22f** |
| Ssa0314BSFU | **H---HAH--AHHHAA-H-AAAAHA--AAAHAHHHAHA-AH** |
| Ssa10044BSFU | **HAAAHAHAHAHHHAAAHA-AAAHA-A-AAHAHHHAHAHAH** |
| Ssa1010BSFU | **HAAAHAHAHAHHHAAAH-AAAAHAAAAAAHAHHHAHAHAH** |
|  |  |
| chrom | **NB1-23af** |
| Ssa1080BSFU | **HAAAAAHHHHAHAAHHAHAHAHAAHAAHHHAAAHAAAAAA** |
| Ssa0355BSFU | **-AAAAAHHHHAHAAHHAHAHAHAAHA-HHHAHAH-AHAAA** |
| Ssa1021BSFU | **HAAAAAHHHHAHAHHHAHAHAHAAAAAHHHAHAHAAHAAA** |
| Ssa1078BSFU | **HAA-AAHHHHAHAHHH-HAHAHA--AAHHHAAAHAAHAAA** |
| Ssa0245BSFU | **HAAA-AHHHH--AHHHA-AHAHAAAAAHHHAAAHAAHA-A** |
| Ssa1066BSFU | **HAAAAAHHHHAHAHHHAHAHAHAAAAAHHHAAAHAAHAAA** |
| Ssa0149BSFU | **HAAAAA-HHHAH-HHHAHAHAHAAAAAHHHA-A-AAHA-A** |
| Ssa0095BSFU | **HAAAAAHHHHAHAHHHAH-HAHAAAAAHHHAAAH-AHAAA** |
| Ssa0045ECIG | **HAAAAA-HHHAHAHHHAHAHAHAAAAAHHHAAAHAAHAAA** |
| Ssa0668BSFU | **-AAAAA---HAH-HHHAHAH-HAAAAAHHH--AHAAHAAA** |
| Ssa0710BSFU | **HAAAAAHHHHAHAHHHAHAHAHAHHAAHHHAAAHAHHAAA** |
|  |  |
| chrom | **NB1-23bf** |
| Ssa0287BSFU | **AAAAAHHHHHHHHAAAHHAAAHAHHAAHAAHAAAAAAAAH** |
| Ssa0316BSFU | **---AAHHHHHHHHAAAHHAAAHAHHAAHAAHAAAAAAAAH** |
|  |  |
| chrom | **NB1-24f** |
| Ssa0178BSFU | **HA-AHHAHHHHHHHAAAHHHAAAHAHHAHAAAAAA-AAAH** |
| Ssa0023BSFU | **HAAAHHAAAHHAHHAAAHAHAAAHAHHAHAAAAAAAAHAH** |
| Ssa0874BSFU | **HHHAHHAAAHAAHHHAHHAH-AAHAHHA-AAAAAAAAHAH** |
| Ssa0268BSFU | **HHHAHHAAAHAAHHHAHHAHAAAHAHHAHAAAAAAAAHAH** |
| Ssa0032BSFU | **HHHAHHAAAHAAHHHAHHAAAAAHAHHAHAAAAAAAAHAH** |
| Ssa0485BSFU | **HHHA-HAAA-AAHHHAHAAA-AAHAHHAHAA-AAAAAHAH** |
| Ssa0291BSFU | **HHHAHHAAAHAAHHHAHAAAAAAHAHHAHAAAA-AAAHAH** |
| Ssa0037BSFU | **HHHAHHHA-HAAHHHAAHHAAAAHAHHHHAAAAAAAAHAH** |
| Ssa0232BSFU | **HHHAHHHAAHAHHHAAAHHAAAAHHHAH-AAAAAAAAHHH** |
| Ssa1042BSFU | **HAHAHHHAAHAHHHAAAHHAAAAHHHAHAAAAAAAA-HHH** |
|  |  |
|  |  |
|  |  |
|  |  |
|  |  |

| chrom | **NB1-25af** |
| --- | --- |
| Ssa0293BSFU | **AAHHHHAA-AHAAAHAAAAAHHA-HAH-H-H---AAHAA-** |
| Ssa1000BSFU | **HAHHHHA-HAHAAAHAAAAAHHAHHAHHHHHA----HAAH** |
| Ssa0997BSFU | **HA-HHHAA-AHAAAHAAAAAHHAHHAHHHHHAAAAAHAAH** |
| Ssa0993BSFU | **HAHHHHAAHAHAAAHA-AAAHHAH-AHHHH-A-AAAHAAH** |
| Ssa0992BSFU | **HAHHHHAAHAHAAAHAAAAAHHAHHAHHHHHAAAAAHAAH** |
| Ssa0667BSFU | **-AHH-HAAHAHAAAHAAAAAHHAHHAHHHHHAAAAAHAAH** |
|  |  |
| chrom | **NB1-25bf** |
| Ssa0011BSFU | **A-AAAAHHHAA-HAAAAHHHAA-AA-AAAAHHAAHHHHAH** |
| Ssa0580BSFU | **AHAA-AHHHAAHHAAAAHHHAAHAAAAAAAHHAAHHHHAH** |
| Ssa0375BSFU | **AHAAAAHHHAAHHAAAAHHHAAHAAAAAAAHHAAHHHHAH** |
| Ssa27EVMS | **AHAAAAHHHAAHHAAAHHHHAAHAAAAAHAHHAAHHHHAH** |
| Ssa0298BSFU | **AH-AAAH--AAH----H---AAH---AA----AA-HH-A-** |
| Ssa0275BSFU | **AHAAAAHHHAAHHAAAHHHHAAHAAAAAHAHHAAHHHAAH** |
| Ssa0288BSFU | **-HAAAAHHHAAHHAA-HHHHAAHAAAAAHAHHAAHHHAAH** |
| Ssa0089BSFU | **AHAAAAHHHAAHHAAAHHHHAAHAAAAAHAHHAAHHHAAH** |
| Ssa10137BSFU | **AHAAAAHHHAAHHAAAHHHH-AHAAAAAHAHHAAH-HAAH** |
| Ssa0400BSFU | **--AAAAHHHAAHHAAAHHHH-AHAAA-AHAHHAAHHHAAH** |
|  |  |
| chrom | **NB1-28f** |
| Ssa0613BSFU | **AA---HAAAAAAH-AHHAAHHHAAA-AAHAAAHHAHHAA-** |
| Ssa0257BSFU | **AAHAAHAA-AAHHHAHHAAHHHAAAHAAHAAAHHAHHAAH** |
|  |  |
| chrom | **NB1-31f** |
| Ssa0052BSFU | **AHAAHHHAHHAHAAAAHAAAAAAAHAHHAAHAAAHHAHHH** |
| Ssa0443BSFU | **-HAAAHHAHHAHAAAAAAAHAHAAHAAHAAHAHAHHAHHA** |
| Ssa0172ECIG | **AAAHHHHAHHAHAAAAAAAHAHAAHAAHAAHAHAHHAHHA** |
| Ssa0637BSFU | **AAAHHHAHHHAHAAA-AHAHAHAHAHAAAAHAHAAHAHHA** |
|  |  |
| chrom | **NB1-32f** |
| Ssa0175BSFU | **AHAAHHAAAHAHHAAHHAAHAHAHAHHHAAHAHAHHAHAH** |
| Ssa0161BSFU | **AHAAHHAAAHAHHAAHH-AHAHAHAHHHAAHAHAHHAHAA** |
| Ssa10021BSFU | **-HAAHHAAAHA--AAHHA-HAHAHAHHHAAHAHAHHAHAA** |
| Ssa10022BSFU | **AHAAHHAAAHAHHAAHHAAHAHAHAHHHAAHAHAHHAH-A** |
| Ssa0159BSFU | **AH-AHHAAAHAHHAAHHAAHAHAHAHHHAAHAHAHHAHAA** |
| Ssa0162BSFU | **AH-AHHAAAHAHHAAHHAAHAHAHAHHHAAHAHAHHA-A-** |
| Ssa65 | **AHAAHHAAAHHHHAAHHAAHAHAHAHAHAAHAHAHHAHAA** |
|  |  |
| chrom | **NB1-33f** |
| Saa0099ECIG | **H--A-AAAHAAHA----H-HH-A--A--A-H---A-A--A** |
| Ssa0270BSFU | **HHAAAAAAHAAHAAAHAHHHHHAHHAHAAA-AAAA-AHH-** |
