# Supplementary material for: Comparative genomic analysis of Atlantic salmon, Salmo salar, from Europe and North America
Source: BMC Genet. 2010 Nov 23;11:105. doi: 10.1186/1471-2156-11-105 (PMC2995484; doi:10.1186/1471-2156-11-105)
Supplement: Additional file 7 — Table S3 Phase files for NB1male-specific linkage groups. [file 1471-2156-11-105-S7.DOCX]

| chrom | **NB1-1m** |
| --- | --- |
| Ssa0055BSFU | **HHBBHHHBBBHHHBBBBHHBHBHBBBHBBBHBHHHBHBBH** |
| Ssa0233BSFU | **HH-BHHH-BBHHHBBBBHHBHBHBBBHBBBHBHHHBHBBH** |
| Ssa0057BSFU | **HHBBHHHBBBHHHBBBBHHBHBHBBB-BB-HBHHHBHBBH** |
| SS12 | **HHBBHHHBBBH--BBBBHH-HBHBBBHBBBHBHHHBHBBH** |
| Ssa0183BSFU | **HHBBHHHBBBHHHBBBBHHBHBHBBBHBBBHBHHHBHBBH** |
| Ssa1077BSFU | **HHBBHHHBBBHHHBBBBHHBHBHBBBHBBB-BHHHBHBBH** |
|  |  |
| chrom | **NB1-2m** |
| Ssa1007BSFU | **HHBBBHBBHHBBHBBBBBHHBBBBH-HHBBBHBBBHBBBH** |
| Ssa0844BSFU | **HHBBBHBBHHBB---------BBBHBHHBBBHBBBHBBBH** |
| Ssa0003BSFU | **HHBBBHBBHHBBHBBBBBHHBBBBHBHHBBBHBBBHBBBH** |
| Ssa0026BSFU | **HHBBBHBB-H-BHBBBBBHHBBBBH-HHBBBHBBBHBBBH** |
| Ssa0290BSFU | **HHBBBHBBHHBBHBBBBBHHBBBBHBHHBBBH-BBHBBBH** |
| Ssa1006BSFU | **HHBBBHBBHHBBHBBB-BHHBBBBHBHHBBBHBBBHBBBH** |
| Ssa0031BSFU | **HHBBBHBBHHBBHBBBBBHHBBBB--HHBBBHBBBHBBB-** |
| Ssa0112BSFU | **HHBBBHBBHHBBHBBBBBHHBBBBHBHHBBBHBBBHBBBH** |
| Ssa1086BSFU | **HHBBBHBBHHBBHBBBBBHHBBBB-BHHBBBHBBBHBBBH** |
| Ssa0221BSFU | **HHBBBHBBHHBBHBBBBBHHBBBBHBHH-BBHBBBHBBBH** |
| Ssa1062BSFU | **-H-BBHBBHHBBHBBBBBHHBBBBHBHHBBBHBBBHBBBH** |
|  |  |
| chrom | **NB1-3m** |
| Ssa0377BSFU | **BH---B-HB---H-HHBH--H-HHB--H-BB---HHHHB-** |
| Ssa0273BSFU | **BHBBBBBHBBBHHBHHBHBHHHHHBHBHBBBBHBHHHHBB** |
| SSsp1606 | **BHBBBBBHBBBHHBHHBHBHHHHHBHBHBBBBHBHHHHBB** |
| Ssa0768BSFU | **BHBBBBBHBBBHHBHHBHBHHHHHBHBHBBBBHBHHHHBB** |
| Ssa0153BSFU | **BHBBBBBHBBBHHBHHBHBHHHHHBHBHBBBBHBHHHHBB** |
| Ssa1027BSFU | **BHBBBBBHBBB--BHHBHBHHHHHBHBHBBBBHBHHHHBB** |
| Ssa0863BSFU | **BHBBBBBHBB-HHBHHBHBHHHHHBHBHBBBBHBHHHHBB** |
| Ssa0518BSFU | **BHBBBBBHBBB--BHHBHBHHHHHBHBHBBBBHBHHHHBB** |
|  |  |
| chrom | **NB1-4/32m** |
| Ssa0142BSFU | **HBHBBBHBHBBBBHHBBBHBHHHHBBBHBBBBBHBBHBBB** |
| BHMS376 | **HBHBBBHBHBBBBHHBBBHBHHHHBBBHBBBBBHBBHBBB** |
| BX890355 | **HBHBBBHBHBBBBHHBBBHBHHHHBBBHBBBBBHBBHBBB** |
| Ssa0215BSFU | **HBHBBBHBHBBBBHHBBBHBHHHHBBBHBBBBBHBBHBB-** |
| OMM5037/I | **H-HBBBHBHBBBBHHBBBHB-HHHBBBHBBBBBHBBHBBB** |
| Ssa0376BSFU | **BBHB-BHBHBBBBHHBBBHB-HHHBB-HBBBBBH-B-BBB** |
| Ssa0043BSFU | **BBHBBBHBHB-BBH-BBBHBHBBHBBBHBBBBBHBBHBBB** |
| Ssa0159BSFU | **BB-BBBHBHBHBBHHBBBHBHBHBHBBBBBBBBHBBBBBH** |
| Ssa10022BSFU | **BBHBBBHBHBHBBHHHHBHBHBHBHBBBBBHBBHHHBB-H** |
| Ssa0161BSFU | **BBHBBBHBHBHHBHHHH-HHHBHBHBBHBBHBBHHHBBBH** |
| Ssa65 | **BBHBBBHBHBHHBHHHHBHHHBHBHBBHBBHBBHHHBBBH** |
| Ssa0010BSFU | **BBHBBBHBHBHHBHH--BHHHBHBH-BHBBHBBH-HBBBH** |
| Ssa419 | **BBHBBBHBHBHHBHHHHBHH-BHBHBBHBBHBBHHHBBB-** |
| Ssa1046BSFU | **BBHBBBHBHBHHBHHHHBHHHBHBHB-HBBHBBHHHBBBH** |
| Ssa0162BSFU | **BB-BBBHBHBHHBHHHHBHHHBHBHBBHBBHBBHHHB-B-** |
| Ssa0054BSFU | **BBHBBBHBHBHHBHHHHBHHHBHBHBBHBBHBBHHHBBBH** |
|  |  |

| chrom | **NB1-5m** |
| --- | --- |
| Ssa0826BSFU | **HBHBHBHBHBBHHHBBBBHBBBBHHH-HBBBBHHBHHBBH** |
| Ssa0684BSFU | **HBHBHBHBHBBHHHBHBBHBBBBHHH-HB---HHBHHBBH** |
| Ssa0286BSFU | **HB-BH----B-HHH-HBB-B-BB--H-HB--BHHBH--B-** |
| Ssa0008BSFU | **-BHBHBHBHBB--HBHBBH--BBHH-H-BBBBHHBHHBBH** |
| SSOSL439 | **HBHBHBHBHBBHHHBH-BHB-BBHHHBHBBBBHHBHHBBH** |
| Ssa0488BSFU | **HBHBHBHBHBBHHHBHBBHBBBBHHHBHBBBBHHBHHBBH** |
| Ssa0092BSFU | **HBHBHBHBHBBHHHBHBBHBBBBHHHBHBBBBHHBHHBBH** |
| Ssa0827BSFU | **HBHBHBHBHBBHHHBHBBHB-BBHHHBHBBBB-HBHHBBH** |
| Ssa0941BSFU | **HBHBHB-BHBBH-HBHBBHBBBBHHHB-BBBBHHBHHBBH** |
| Ssa0105BSFU | **HBHBHBHBHBBHHHBHBBHBBBBHHHBHBBBBHHBHHBBH** |
| Ssa0338BSFU | **HBHB-BHBHB-HHHBHBBHBBBB-HHBHBBBBHH-HHBB-** |
| Ssa10035BSFU | **HBHBHBHBHBBHHHBHBBHBBBBHHHBBBBBBHHBHHBBH** |
|  |  |
| chrom | **NB1-6m** |
| Ssa0825BSFU | **BBBHHBBBBBBB-BHHHBBH-HHBBB-HBBHHHH-BHBBH** |
| Ssa0208BSFU | **BBBH-BBBBBBBBBHHHBBHHHHBBBHHBBHHHHHBHBBH** |
| Ssa0278BSFU | **BBBHHBBBBBBBBBHHHBBHHHHBBBHHBBHHHHHBHBBH** |
| Ssa0196BSFU | **BBBHHBBBBBBBBBHHHBBHHHHBBBHHBBHHHHHBHBBH** |
| Ssa0554BSFU | **BBBHHBBBBBBBBBHHHBBHHHHBBBHHBBHHHHHBHBBH** |
| Ssa0098BSFU | **BBBHHBBBBBBBBB-HHBBHHHHBBBHHBBHHHHHBHBBH** |
| Ssa0835BSFU | **HBBHHBBBBBBBBBHHHBBHHHHBBBHHBBHHHHHBHBBH** |
|  |  |
| chrom | **NB1-7m** |
| Ssa0937BSFU | **BHHBHHHHBHHBBHHBBHBB-BBBHB-BBHHBB-HBBHBB** |
| Ssa0107BSFU | **-HHB-HHHBHHBBHH-BH-BHBBBHBHBBHH-BHHBBHBB** |
| Ssa10023BSFU | **BHHBHHHHBHHBBHHBBHBBHBBBHBHBBHHBBHHBBHBB** |
| Ssa0006BSFU | **BHHB-HHHBHH-BHHBBHB-HBBBH--BBHHBB--BBHBB** |
| Ssa0132BSFU | **BH-BHHHH-HHBBHHBBHBB-BBBHBHB-HHBBHHBBHBB** |
|  |  |
| chrom | **NB1-8m** |
| Ssa0259BSFU | **HBBHHHBBBHHHBBHHBHBHHBBBBBBHHHHHHBBB-BHH** |
| Ssa0226BSFU | **HBBHHHBBBHHHBBHHBHBHHBH-BBBHBBBHHBBBHBHH** |
| Ssa0957BSFU | **-BBHB-B-BHHHBBHHBHBHHBHBBBB-BBBHHBBBHBHH** |
| Ssa0218ECIG | **-BBH-H----H-BBH----H-B--B-BH-B-H-B--HBHH** |
| Ssa0051BSFU | **HBBHBHBBBHHHBBHHBHBHHBHBBBBHBBBHHBBBHBHH** |
| Ssa0227BSFU | **HBBHBHBBBHHHBBHHBHBHHBHBBBBHBBBHHBBBHBHH** |
| Ssa0050BSFU | **HBBHBHBBBHH-BBHHBHBHH-HBBBB-BBBHHBBBHBH-** |
| Ssa0301BSFU | **HBBHBHBBBHHHBBHHBHBHHBHBBBBHBBBHHBBBHBHH** |
| Ssa0666BSFU | **HBBHBHBBBHHHBBHHBHBHHBHBBBBHBBBHHBBBHBHH** |
| Ssa0918BSFU | **HBBHBHBBBHHHBBHHBHBHHBHBBBBHBBBHHBBBHBHH** |
| Ssa197DU | **HBBHBHBBBHHHBBHHBHHHHBHBBBBHBBBHHBHHH-HH** |
|  |  |

| chrom | **NB1-9m** |
| --- | --- |
| Ssa0503BSFU | **-HHBBHH-HHBB-BHBBBBH-HBHBH-BHHHHBH-BBHBH** |
| Ssa1052BSFU | **HHHBBHHBHHBHBBHBBBBHBHBHBHBBHHHHBHBBBHBH** |
| Ssa0216BSFU | **HHHBBHH-HHBHBBHB-BBHB-BHBHBBHHHHBHBBBHBH** |
| Ssa0229BSFU | **HHHBBHHBHHBHBBHBBBBHBHBHBHBBHH-HBHBBBHBH** |
| Ssa0005BSFU | **-HHBBHHBHHBHBBHBBBBHBHBHB-BBHHHHB-BBBHBH** |
| Ssa0122BSFU | **HHHBBHHBHHB-BB-BBB-HBHBHBHBBHHHHBHBBBHBH** |
| Ssa0091BSFU | **HHHBBHHBHHBHBBHBBBB-BHBHBHBBHHHHBHBBBHBH** |
| Ssa0254BSFU | **HHHBBHHBHHBHBBHBBBBHBHBHBHBBHHHHBHBBBHBH** |
| Ssa0819BSFU | **HHHBBHHBHHBHBBHBBBBHBHBHBHBBHHHHBHBBBHBH** |
| Ssa0028ECIG | **----BH-B--BH---BBBBH-HB--H-B-H-HBHB--H-H** |
| Ssa0692BSFU | **HHHBBHHBHHBHBBHBBBBHBHBHBHBBHHHHBHBBBHBH** |
| Ssa0902BSFU | **-HHBBHHBHHBHBBHBBBBH-HBHBHBBHHHHBHBBBHBH** |
| SSsp3016 | **-HHBBH-BHHBHBB-BB-BHBHBHB-BBHHHHB-BBBHBH** |
| Ssa1016BSFU | **HH-BBHHBHHBHBBHBBBBHBHBH-HBBHHHHBHBBBHBH** |
|  |  |
| chrom | **NB1-10am** |
| Ssa0885BSFU | **HBHHB-HBHHBBHBHBHBBBHBBHHHHBBBBBHBHHHBBH** |
| Ssa0815BSFU | **HBHHBHHBH-BBHB-BHBBB-BBHHHHBBBBBHBHHHBBH** |
| Ssa0038BSFU | **HBHHBHHB-HBB-BHBHBBBHBBHHHHBBBBBHBHHHBBH** |
| Ssa0042BSFU | **HBHHBHHBHHBBHBHBHBBBHBBHHHHBBBBBHBHHHBBH** |
| Ssa1061BSFU | **HBHHBHHB-HBBHBHBHBBBHBBHHHHBBBBBHBHHHBBH** |
| Ssa1070BSFU | **HBHHHHHBHHBBHBHB-BBBHBBHHHHBBBBBHBHHHBBH** |
| Ssa0850BSFU | **HBHHHHHBHHBBHBHBHBBBHBBHHHHBBBBBHHBHHBBH** |
|  |  |
| chrom | **NB1-10bm** |
| SSOSL85 | **B-BBB-BBHHBBBBHBHBBBHBBHBHBBBBBBHBBHHBB-** |
| Ssa10138BSFU | **-BBB-BBBHHBBHBHBHBBB-B-BB-BBBB-B-BBHBBBB** |
|  |  |
| chrom | **NB1-11m** |
| Ssa0670BSFU | **BHHHHBBBBBBBHBBBBBBHBHHBHBBHBHBHBHBHHBHB** |
| SsaD144 | **BHHHHBHBBBBBHBBBBBBHBHHBHBBHBHBHBHBHHBHB** |
| Ssa0017BSFU | **BHHHHBHBBBBBHBBBBBBHBHHBHBBHBHBHBHBHHBHB** |
| Ssleel53 | **BHHHHBHBBB-BHBBBBBBHBHHBHBBHBHBHBHB-HBH-** |
| Ssa0093BSFU | **BHHHHBHBBBBBHBBBBBBHBHHBHBB-BHBH-HBHHBHB** |
| Ssa0069BSFU | **BBBHBBHBBBBBHBBBBBBHBHHBHB-HBHBHBHB-HBHB** |
|  |  |
| chrom | **NB1-12m** |
| Ssa0808BSFU | **BHHHBBBHHBBB-BHBHBHHBBBHHBHBBBBBHHBBHBHH** |
| Ssa0571BSFU | **BHHHBBBHHBBBHHHBHBHHBBHHBBHBBBBBHHBBHBHH** |
| Ssa0063BSFU/I | **BHHHBBBHHBBBHHHBHBHHBBHHBBHBBBBBHHBBHBHH** |
| Ssa0064BSFU | **BHHHBBBHHBBBHHHBHBHHBBHHBBHBBBBBHHBBHBHH** |
| Ssa0897BSFU | **BHHHBBBHHBBBHHHBHBHHBBHHBBHBBBBBHH-BHBHH** |
|  |  |
| chrom | **NB1-13m** |
| Ssa0138BSFU | **BBHHBBHHHHH-HBHHBB-BBBHHBBHHBHHBHHBBBBBB** |
| Ssa0816BSFU | **BBHHBBHHHHHBHBHHBBHB-BHHBBHHBHHBHHBBBBBB** |
| Ssa164 | **BBHH-BHHHHHBHBHHBBHBBBHHBBHHBHHBHHBBBBBB** |
| Ssa0609BSFU | **BBHHBBHHHHHBHBHHBBHBBBHHBBHHBHHBHHBBBBBB** |
| Ssa0308BSFU | **---HB-HHHH---BH-BBHB--H--B-HB-HBHHBBBB--** |
| Ssa0823BSFU | **BBHHBBHHHHHBHBHHBBHBBBHHBBBHBHHBHHBBBBBB** |
| Ssa0265BSFU | **BBHHBBHHHHHBHBHHBBHBBBHHBBB-BHHBHHBBBBBB** |
|  |  |

| chrom | **NB1-14m** |
| --- | --- |
| Ssa0689BSFU | **HHBBHHBHBBHBHHHBBBBBBBBH-HBBBBBHHHHHBHHH** |
| Ssa1018BSFU | **HHBBHBBHBBHBHHHBBBBBBBBHHHBBBB-HHHHHBHHH** |
|  |  |
| chrom | **NB1-15m** |
| Ssa0984BSFU | **BBBBHBBHBBBH-HBB-HBHBHH--BBHBBHBBBHBBB-B** |
| Ssa1034BSFU | **HBBBHBBHBBBH-HBBHHBHBHHBHBBHBBHBBBHBBBBB** |
| Ssa1067BSFU | **HBBBHBBH-BBHHHBBHHBHBHHB-BBHBBHBBBHBBBBB** |
| Ssa1038BSFU | **HBBBHBBHBBBHHHBBHHBHBHHBHBBHBBHBBBHBBBBB** |
| Ssa1036BSFU | **HBBBHBBHBBBHHHBBHHBHBHHBHBBHBBHBBBHBBBBB** |
| Ssa0165BSFU | **HBBBHBBHBBBHHHBBHHBHBHHBHBBHBBHBBBHBBBBB** |
| Ssa1035BSFU | **HBBBHBBHBBBHHHBBHHBHBHHBHBBHBBHBBBHBBBBB** |
| Ssa0205ECIG | **HBBBHBBHBBBHHHBBHHBHBHHBHBBHBBHBBBHBBBBB** |
| BHMS127 | **HBBBHBB-BBBHHBBBHHBBBHHBHBBHBBHBBBHBBBBH** |
|  |  |
| chrom | **NB1-16m** |
| Ssa1083BSFU | **HHHBBHHHBBBHBBBBBHBBHBH-HBBHHBBBHHBBBHHH** |
| Ssa0184BSFU | **HHHBBHHHHBBHBBBBBHBBHBHBHBBHHBBBHHBBBHHH** |
| Ssa0125BSFU | **HHHBBHHHHBBHBBBBBHBBHBHBHBBHHBB-HHBBBHHH** |
| Ssa0192BSFU | **HHHBBHHHHBBHBBBBBHBBHBHBHBBHHBBBHHBBBHHH** |
| Ssa0307BSFU | **HHHBBHHHHB--BBB---B-HBHBHBBHHBBBHHBB---H** |
| Ssa0206BSFU | **HHHBBHHHHBBHBBBBBHBBHBHBHBBHHBBBHHBBBHHH** |
| Ssa0156BSFU | **HH-BBHHHHBBHBBBBBHBBHBHBHBBHHBBB-HBBBHHH** |
| Ssa0207ECIG | **HHHBBH--HBBHBBBBBHBBHBHBHBB-HBBBHHBBBHHH** |
| Ssa0067BSFU/I | **HHHB-HHHHBBHBBBBBHBBHBHBHBBHHBBBHHBBBHHH** |
| Ssa0249BSFU | **HHHBB-HH--BHBBBBBHBBHBHBHBBHHBBBHHBBBHHH** |
| Ssa0462BSFU | **-HHBBHHHHBBH-BBBBHBB-BHBHBBHHBBBHH-BBHHH** |
| Ssa0030BSFU | **BHHBBHHHHBBHBBBBBHBBHBHBHBBHHBBBHHBBBHHH** |
|  |  |
| chrom | **NB1-17m** |
| Ssa0171BSFU | **HHHHHHHHBHBBHBBHHBHH-HHBBBBH--BBBH-HBHBB** |
| Ssa1005BSFU | **HHHHHHHBBHBBHBBHHBHHHHHBBBBHHBBBBHHHBBBB** |
| Ssa0033BSFU | **HHHHH-HBBHBBHBBHHBHHHHHBBBBHHBBBBHHHBBBB** |
| Ssa0173BSFU | **HHHHHHHBBHBBHBBHHBHHHHHBBBBHHBBBBHHHBBBB** |
| Ssa0306BSFU | **HHBHHHHBBHBBHBBHHBHHHHHBBBBHHBBBBHHHBBBB** |
| Ssa0067BSFU/II | **HHBH-HHBBHBBHBBHHBHHHHHBBBBHHBBBBHHHBBBB** |
| Ssa0309BSFU | **---H--HBB-BB-BB-HBHHHHHBBBBHHBBBBHHHBBBB** |
| Ssa0246BSFU | **HHBHHHHBBHBBHBBHHBH-HHHBBBBHHBBBBHH--BBB** |
| Ssa1043BSFU | **HHBHHHHBBHBBHBBHHBHHHHHBBBBHHBBBBHHHBBBB** |
| Ssa0039BSFU | **-HBHHHHBBHBBHBBHHBHHHHHBBBBHHB-BBHHHBBBB** |
| Ssa0016BSFU | **HHBHHHHBBHBBHBBHHBHHHHHBBBBHHBBBBHHHBBBB** |
| Ssa0630BSFU | **HHBHHHHBBHBBHBBHHBHHHHHBBBBHHBBBBHHHBBBB** |
| Ssa0002BSFU | **BHB--HHHBHBBHBBHHBHHHHB-BBBHHBBBBH--BBBB** |
|  |  |
| chrom | **NB1-18m** |
| Ssa0072BSFU | **BHHBBHHBHBBBBBBBBBHBBHHBBBBHBBHBBBHHBHHB** |
| Ssa0295BSFU | **BH-BBH---BBBBBBBBBHHBHH-BBBHBBHBBBHH---B** |
| Ssa0261BSFU | **BHHBBHHBHBBBBBBBBBHHBHHBBBBHBBHBBBHHBHHB** |
| Ssa0203BSFU | **BHHBBHHBHBBBBBBBBBHHBHHBBBBHBBHBBBHHBHHB** |
| Ssa0102BSFU | **BHHBB-HBHBBBBBBBBBHHBHHBBB-HB-HBBBHHBHHB** |
| Ssa0328BSFU | **B-HBBH-B-BBBBBBBBB--BHHBBBBHBB-BBBHHBHHB** |
| Ssa87 | **BHHBBHHBHBBBBBBBBBHHBHHBBBBHBBHBBBHHBHHB** |
| Ssa0665BSFU | **BHHBBHHBHBBBBBBBBBHHBH-BBBBHBBHBBBHHBHHB** |
| Ssa0052ECIG | **BH-BBHHB----BBBB-BHHB---B-B-B-HBBB--B-HB** |
|  |  |
| chrom | **NB1-19m** |
| Ssa0604BSFU | **HBBBHHBHHBBHBBHBHHBBBHBHHBHHBBBHBBBHBHBB** |
| Ssa1074BSFU | **HBBBHHBH-BBHBBHBHHBBBHBHHBHHBBBHBBBHBHBB** |
|  |  |
| chrom | **NB1-20m** |
| Ssa0733BSFU | **HBBBBHBBBHBHHHHHBBHBHHHBHHBBBHHBBHBBHHBH** |
| Ssa0686BSFU | **-B-BB-BBBHBHHHHHBBHBHHHBHHBBBHHBBHBBHHBH** |
| Ssa0317BSFU | **HBBBBHBBBHBHHHHHBBHBHHHBHHBBBHHBBHBBHHBH** |
| Ssa0872BSFU | **HBBBBHBBBHBHHHHHBBHBHHHBHHBBBHHBBHBBHHBH** |
| Ssa0585BSFU | **HBBBBHBBBHB-HHHHBBHBHHHBHHBBBHHBBHBBHHBH** |
| Ssa0283BSFU | **-BBBBHBBBHBHHHHHBBHBHHHBHHBBBHHBBHBBHHBH** |
|  |  |
| chrom | **NB1-21/33m** |
| Ssa0868BSFU | **HBHBHBBBHB-HBHHBBHHHHBBHBBBBBHHHHHBHBHHH** |
| Saa0099ECIG | **H--B-BBBHBBHB----H-HH-B--B--B-H---B-B--B** |
| Ssa0001BSFU | **-BHBHBBBHBBHBHHBBHBHHB-HBBBBBHHHHH-HBBHB** |
| Ssa0735BSFU | **HBHBHBBBHBBHBHHBBHBHHBBHBBBBBHHHHHBHBHHB** |
| Ssa0323BSFU | **HBHBHBBBHBBHBHHBBHBHHBBHBBBBBHHHHHBHBHHB** |
| OmyRGT6TUF | **-BHBHBBBHBBHBHHBBHBHHB-HBBBBBHHHHHBHBHHB** |
| Alu333 | **HBHBHBBBHBBHB-HBBHBHHBBHBBBBBHHHHHBHBHHB** |
| OmyRGT44TUF | **HBHBHBBBHBBHBHHBBHBHHBBHBBBBBHHHHHBHBHHB** |
| Ssa0270BSFU | **HBHBHBBBHBBHBHHBBHBHHBBHBBBBBH-HHHB-BHH-** |
| CA368462 | **HBHBHBBBHBBHBHHBBHBHHBBHBBBBBHHHHHBHBHHB** |
| OMM5149 | **-HBHBB-BHBBHBHHBBHBHHBBHBB-B-H-HHHBHBHHB** |
|  |  |
| chrom | **NB1-22m** |
| Ssa1010BSFU | **BBBHBHBHHBHBHBBHB-BHHHBHHHHHBHBHHBHBBHHB** |
| Ssa0266BSFU | **BBBHBHB-HBHBH-BHBHBHHHBHHHHHBHB-HBHBBHHB** |
| Ssa10044BSFU | **BBBHBHBHHBHBHBBHBH-HHHBH-H-HBHBHHBHBBHHB** |
| Ssa0314BSFU | **B---BBB--BHBHBB-B-BHHHBH--HBBHBHHBBBB-HB** |
|  |  |
| chrom | **NB1-23m** |
| Ssa1021BSFU | **HBBBBBHHHBBHBHBHBHBHBBHBBBBHHBBHBBHBHHHB** |
| Ssa0668BSFU | **-BBBBB---BBH-HBHBHHH-BHBBBBHHB--BBHBHHHB** |
| Ssa1080BSFU | **HBBBBBHHHBBHBHBHBHHHBBHBBBBHHBBHBBHBHHHB** |
| Ssa0287BSFU | **HBBBBBHHHBBHBHBHBHHHBBHBBBBHHBBHBBHBHHHB** |
| Ssa0149BSFU | **HBBBBB-HHBBH-HBHBHHHBBHBBBBHHBB-B-HBHH-B** |
| Ssa1066BSFU | **HBBBBBHHHBBHBHBHBHHHBBHBBBBHHBBHBBHBHHHB** |
| Ssa0245BSFU | **HBBB-BHHHB--BHBHB-HHBBHBBBBHHBBHBBHBHH-B** |
| Ssa1078BSFU | **HBB-BBHHHBBHBHBH-HHHBBH--BBHHBBHBBHBHHHB** |
| Ssa0355BSFU | **-BBBBBHHHBBHBHBHBHHHBBHBBB-HHBBHBB-BHHHB** |
| Ssa0710BSFU | **HBBBBBHHHBBHBHBHBHHHBBHHHBBHHBBHBBHHHHHB** |
|  |  |
| chrom | **NB1-24m** |
| Ssa0230BSFU | **HBHHBHBBBBHBBHBBBBBHBHHHBHBHHHBBHBHBBBB-** |
| Ssa1042BSFU | **HBHHBHBBBBHBBHBBBBBHBHHHBHBHHHBBHBHB-BBB** |
| Ssa0032BSFU | **HBHHBHBBBBHBBHBBBBBHBHHHBHBHHHBBHBHBBBBB** |
| Ssa0485BSFU | **HBHH-HBBB-HBBHBBBBBH-HHHBHBHHHB-HBHBBBBB** |
| Ssa0268BSFU | **HBHHBHBBBBHBBHBBBBBHBHHHBHBHHHBBHBHBBBBB** |
| Ssa0178BSFU | **HB-HBHBBBBHBBHBBBBBHBHHHBHBHHHBBHBH-BBBB** |
| Ssa0023BSFU | **HBHHBHBBBBHBBHBBBBBHBHHHBHBHHHBBHBHBBBBB** |
| Ssa0291BSFU | **HBHHBHBBBBHBBHBHBBBHBHHHBHBHHHBBH-HBHBBB** |
|  |  |
| chrom | **NB1-25m** |
| Ssa0288BSFU | **-HBHBBHBBBHBHHB-BBBHBHBBBBHBBBHBBBHHHHHB** |
| Ssa0298BSFU | **BH-HBBH--BHB----B---BHB---HB----BB-HH-H-** |
| Ssa0275BSFU | **BHBHBBHBBBHBHHHBBBBHBHBHHBHBBHHBBBHHHHHB** |
| Ssa0089BSFU | **BHBHBBHBBBHBHHHBBBBHBHBHHBHBBHHBBBHHHHHB** |
| Ssa0011BSFU | **B-BHBBHBBBH-HHHBBBBHBH-HH-HBBHHBBBHHHHHB** |
| Ssa0293BSFU | **BHBHBBHB-BHBHHHBBBBHBHB-HBH-B-H---HHHHH-** |
| Ssa0524BSFU | **BHBHBBHBBBHBHHHBBBBH-HBHHHHBBHHBBBHHHHHB** |
| Ssa0147SSFU | **BHBHBBHBBBHBHHH-BBBHBHBHHBHBBHHBBB-HHHHB** |
| Ssa0997BSFU | **BH-HBBHB-BHBHHHBBBBHBHBHHBHBBHHBBBHHHHHB** |
| Ssa0037ECIG | **BHBHBBHBBBHBHHHBBBBHBHBHHBHBBHHBBBHHHHHB** |
| Ssa0580BSFU | **BHBH-BHBBBHBHHHBBBBHBHBHHBHBBHHBBBHHHHHB** |
| Ssa0667BSFU | **-HBH-BHBBBHBHHHBBBBHBHBHHBHBBHHBBBHHHHHB** |
| Ssa0993BSFU | **BHBHBBHBBBHBHHHB-BBHBHBH-BHBBH-B-BHHHHHB** |
| Ssa1000BSFU | **BHBHBBH-BBHBHHHBBBBHBBBHHBHBBHHB----HHHB** |
| Ssa0400BSFU | **--BHBBHHBBHBHHHBBBBH-HBHHB-BHHHBBBHHHHHB** |
| Ssa10137BSFU | **BBBHBBHHBBHBHHHBBBBH-HBHHBHBHHHBBBH-HHHB** |
|  |  |
| chrom | **NB1-28m** |
| Ssa0613BSFU | **BH---BBHBHHBH-BHBHHBHBBHH-BHBHBBHBBBBBB-** |
| Ssa0013BSFU | **BHBHHBBHBHHHHBBHBHHBHBBHHHBHBHBBHBBBBBBH** |
| Ssa0257BSFU | **BHBHHBBH-HHHHBBHBHHBHBBHHHBHBHBBHBBBBBBH** |
|  |  |
| chrom | **NB1-31m** |
| Ssa0052BSFU | **HHHBHHHHBBHHBBBBBHBHBBHHBHHHBBBBBHHBHHBB** |
| Ssa0637BSFU | **HHHBBHBHBBHHBBB-BHHHBBHHBHHHBBBBBHHBHHBB** |
| Ssa0421BSFU | **HHHBBHBHBBHHBBBBBHHHBBHHBHHHBBBBBHHBHHBB** |
| Ssa0817BSFU | **HHHBBHBHBBHHBBBBBHHHBBHHBHHHBBBBBBHHBHBB** |
